# Evaluating the Efficacy of Internet-Delivered Cognitive Behavioral Therapy Blended With Synchronous Chat Sessions to Treat Adolescent Depression: Randomized Controlled Trial

**DOI:** 10.2196/13393

**Published:** 2019-11-01

**Authors:** Naira Topooco, Sandra Byléhn, Ellen Dahlström Nysäter, Jenny Holmlund, Johanna Lindegaard, Sanna Johansson, Linnea Åberg, Lise Bergman Nordgren, Maria Zetterqvist, Gerhard Andersson

**Affiliations:** 1 Department of Behavioural Sciences and Learning Linköping University Linköping Sweden; 2 Center for m^2^Health Palo Alto, CA United States; 3 Centre for Psychiatry Research Department of Clinical Neuroscience Karolinska Institutet Stockholm Sweden; 4 Center for Social and Affective Neuroscience Department of Clinical and Experimental Medicine Linköping University Linköping Sweden; 5 Department of Child and Adolescent Psychiatry Region Östergötland Linköping Sweden

**Keywords:** adolescent, depression, cognitive behavioral therapy, randomized controlled trial, internet, digital health, technology, mental health, text messaging, instant messaging

## Abstract

**Background:**

Depression is a common and serious problem among adolescents, but few seek or have access to therapy. Internet-delivered cognitive behavioral therapies (ICBTs), developed to increase treatment access, show promise in reducing depression. The inclusion of coach support in treatment is desired and may be needed.

**Objective:**

The aim of this study was to determine the efficacy of an ICBT protocol blended with weekly real-time therapist sessions via chat; blended treatment, for adolescent depression, including major depressive episode (MDE). The protocol has previously been evaluated in a controlled study.

**Methods:**

In a two-arm randomized controlled trial, adolescents 15 to 19 years of age were recruited through a community setting at the national level in Sweden (n=70) and allocated to either 8 weeks of treatment or to minimal attention control. Depression was assessed at baseline, at posttreatment, and at 12 months following treatment (in the intervention group). The primary outcome was self-reported depression level as measured with the Beck Depression Inventory II at posttreatment. The intervention was offered without the need for parental consent.

**Results:**

Over two weeks, 162 adolescents registered and completed the baseline screening. Eligible participants (n=70) were on average 17.5 years of age (SD 1.15), female (96%, 67/70), suffered from MDE (76%, 53/70), had no previous treatment experience (64%, 45/70), and reported guardian(s) to be aware about their depression state (71%, 50/70). The average intervention completion was 74% (11.8 of 16 modules and sessions). Following the treatment, ICBT participants demonstrated a significant decrease in depression symptoms compared with controls (*P*<.001), corresponding to a large between-group effect (intention-to-treat analysis: *d*=0.86, 95% CI 0.37-1.35; of completer analysis: *d*=0.99, 95% CI 0.48-1.51). A significant between-group effect was observed in the secondary depression outcome (*P*=.003); clinically significant improvement was found in 46% (16/35) of ICBT participants compared with 11% (4/35) in the control group (*P*=.001).

**Conclusions:**

The results are in line with our previous study, further demonstrating that adolescents with depression can successfully be engaged in and experience significant improvement following ICBT blended with therapist chat sessions. Findings on participants’ age and baseline depression severity are of interest in relation to used study methods.

**Trial Registration:**

ClinicalTrials.gov NCT02363205; https://clinicaltrials.gov/ct2/show/NCT02363205

## Introduction

### Background

Unipolar depressive disorders are the leading cause of disability-adjusted life years among adolescents aged 15 to 19 years globally [1]. Early age of onset of depression is a risk factor for recurrent depression [2,3], is associated with poor academic achievement, unemployment, and impaired quality of life [2,4-6] and predicts additional and worsened mental and physical illness [7-9]. Despite the high level of disability, only a small fraction of young people in need have access to any kind of intervention [10,11].

### Internet-Delivered Psychological Treatment

At this time, psychological treatment in the form of cognitive behavioral therapy (CBT) is considered one of the best empirically supported behavioral interventions to reduce depression [12-15]. The theoretical framework of CBT is rooted in the central assumption that depression is caused and maintained by unhelpful cognitions and behaviors, with treatment accordingly focused on improving function in these domains with the application of skill-based behavioral strategies [16,17]. Worldwide, adaptions of in-person psychotherapy protocols into Web-based formats, the majority involving CBT, are emerging to bring mental health interventions to individuals who for different reasons are not reached by regular services. In internet-delivered CBT, often referred to as ICBT, content in the form of text, video, or audio is arranged into weekly *modules* or *sessions* and delivered inside a Web-based treatment platform along with homework assignments [18]. Programs are offered as self-help or involve a clinician with the role of guiding the participant through the program and providing brief feedback on treatment progress. Clinician support is often provided asynchronously (eg, email), with the administration time typically not extending 20 min per participant and week [19].

### The Role of Support in Internet-Delivered Cognitive Behavioral Therapy

Concerning ICBT and other computer and Web-based interventions for depression, one of the most consistent findings is that human support matters. The social element—having someone to talk to or just knowing that someone is there monitoring and listening, seems important in maintaining motivation to continue treatment and experiencing improvement from it. Although there are exceptions in the literature (eg [20]), coached programs are shown to be more effective than self-guided programs in the treatment of adult depression [21-23] with guided ICBT demonstrating effects similar to those found with in-person CBT [24,25]. In comparison, self-guided ICBTs produce small treatment effects that sometimes merely surpass the lower cut-off point for what is considered clinical relevance in the treatment of depression [26,27]. *True* self-help ICBTs, delivered freely to anyone and without any human interaction in connection to the intervention (eg, interview and administrative contact), show among the highest dropout rates and smallest effects in the field [28]. A study on patients’ perspectives on ICBT for depression points out the importance of guidance in making treatment work [29]. Existing ICBT programs produce benefits for youth depression, but there is considerable variation in populations, measures used, and outcomes [11,30-32]. The influence of support on outcomes in computer and Web-based intervention for youth depression and anxiety, however, was recently reported in a meta-analysis by Grist et al [33], in line with results for ICBT with adult populations. On the basis of 34 studies with 3113 children and adolescents, programs that included >90 min supportive contact yielded higher effect sizes (*g*=0.87) than purely self-help programs (*g*=0.24), although interventions based on theoretical frameworks other than CBT were included in the analyses. A review focusing on how Web-based depression programs for adolescents work, including ICBT programs, found that completion rates increased if the treatment was delivered with real-time guidance from a doctor, therapist, or teacher [34]. Even programs that included automated reminders, praise, or suggestions were only able to optimize adherence with real-time, in-person contact. On the individual study level, depression prevention studies with youth have found an almost 10-fold difference in program completion between ICBT when offered with teacher support or monitoring as opposed to self-help [35], and that adolescents report stopping intervention because of the need to talk to someone, rather than doing a program [36]. It is not necessarily a problem that unguided behavioral interventions offered to communities are associated with limited effects—no additional cost is associated with repeated use of the intervention, and despite, for example, high dropout rate, a large number of individuals will still potentially benefit from the interventions. However, for the treatment of clinical depression and in the treatment of youth—a particularly vulnerable population—the apparent ability of coach support to improve outcomes in ICBT warrants consideration of how support can be strengthened and provided to adolescents.

### A Text-Based Blended Treatment Approach

To include a strong therapist interaction in the form of sessions is in line with aggregated findings that support is desired and boosts the effect of ICBT. Indeed, emerging *blended treatment* approaches [37,38] that integrate some in-person therapist sessions with mobile or internet sessions in the same protocol—with the rationale of providing a strong therapist interaction while keeping the advantage of reliable self-help components—show promise in outcomes [39,40] and acceptability [41,42] for adult depression. Among young people, the preference for real-time texting and instant messaging (chat) is well established [43,44]. In the United States, nearly 1 in 3 (32%) of adolescents and young adults with moderate to severe depressive symptoms report having used texting, Web-based messaging, an app, or video chat to connect with a health care provider [45]. To include strong therapist support in the form of sessions (ie, blended treatment), and moreover, doing so with real-time texting is consistent with adolescents’ media use preferences, while maintaining the combination of easy access and discretion offered in ICBT, which for young people may prove especially effective in overcoming barriers to behavioral intervention. The inclusion of strong therapist interaction could moreover extend ICBT to appropriately address clinical depression in youth. In light of full-threshold depressive episodes being commonly experienced in adolescence and young adulthood despite prevention effort [46], this is highly relevant.

### Objective

This study investigated a treatment protocol consisting of ICBT modules blended with weekly therapist chat sessions for adolescent depression, including major depressive episode (MDE). We have previously evaluated the treatment in a controlled trial with promising results (*d*=0.71 against minimal attention control [47]). In line with participant feedback, the protocol was subsequently revised to include longer sessions while the conceptual model of delivery was kept intact. The main objective of this study was to further establish the effect of the treatment model, using comparable eligibility criteria and study methods, including allowing adolescents aged 15 to 17 years to participate without parental involvement. We hypothesized that the intervention would outperform the control condition in reducing depression, corresponding to at least moderate between-group effect.

## Methods

### Study Design and Participants

In a 2-arm randomized controlled trial, adolescents were recruited in a community setting at the national level in Sweden and randomized (1:1 ratio) to ICBT (n=35) or to minimal attention control (n=35). Enrollment and baseline assessments took place between January 26 and February 10, treatments were conducted between February 13 and April 9, and posttreatment assessments (at 8 weeks) were conducted between April 10 and 16, 2017. Controls were given access to treatment following the posttreatment assessment (8 weeks), and ICBT participants were reassessed 12 months following treatment (April 2018). Eligible participants were 15 to 19 years, suffered depressive symptoms (≥14 points on the Beck Depression Inventory II; BDI-II [48]), and presented at least 4 symptoms including 1 core symptom, or fulfilled criteria for major depressive episode (MDE) according to The Mini-International Neuropsychiatric Interview (MINI 7.0 [49]). We excluded individuals who were receiving psychological therapy, were alcohol or drug dependent, showed severe suicidal ideation, or who had severe comorbid psychiatric conditions (eg, bipolar disorder or psychotic symptoms). Comorbid anxiety disorder(s) were allowed if depression was the principle concern. Medication for anxiety, depression, or Attention deficit hyperactivity disorder was accepted if the dose had been stable >1 month before the study. Minors aged 15 to 17 years were included in line with Swedish research legislation, which states that individuals aged 15 to 17 years can consent to research without parental involvement if possessing sufficient maturity to freely undertake study participation with awareness of possible adverse consequences.

Participants were recruited by social media posts from study staff including one guest post in a wide-reaching Instagram account focusing on coping with mental health issues. Posts described the opportunity to receive Web-based psychological treatment within a research study. Information was also distributed to schools, youth centers, and clinics across Sweden. Potential participants were directed to the study website and registered for the study by creating a user account, providing informed consent (checking a box), and completing a Web-based screen (demographics and self-reported outcome measures). An encrypted Web-based treatment platform, *Iterapi*, was used to collect screening data [50]. Individuals who showed initial eligibility were invited to a phone interview with study staff to confirm eligibility using the full MINI, to determine matureness to participate, to obtain verbal consent, and to confirm identity (name, address, and personal identity number). The principal investigator decided on final eligibility. Before the randomization, eligible participants were requested to sign a digital consent sheet (full name with digital date stamp) to confirm their willingness to participate in the study. After consent was agreed and baseline data collected, participants were stratified according to depression severity (fulfilling DSM-5 criteria for MDE or not), and thereafter randomized in a 1:1 ratio. A person not involved in the study executed the randomization procedure using a computer-generated sequence service. Treatment was given open label. The Research Ethics Board in Linköping, Sweden, gave approval for the study (Reg. no. 2014/427-31). The study was registered at ClinicalTrials.gov (NCT02363205). Participants were not offered financial compensation for treatment or assessment completion at any time.

### Interventions: Internet-Delivered Cognitive Behavioral Therapy Program

The treatment included 8 ICBT modules, and 8 individual therapist sessions delivered via chat; the entire intervention lasted 8 weeks. Treatment took place within an encrypted Web-based treatment platform: *Iterapi* [50]. Modules comprised text material and videos, fictional storylines, reflection tasks and homework assignments, and entailed the behavioral and cognitive approach of CBT. Core techniques included *behavioral activation:* detecting unhelpful behavior, and reinstating and reinforcing behaviors that increase positive consequences, thus elevating mood [51,52], and *cognitive restructuring*: correcting maladaptive thinking patterns and inaccurate beliefs (negatively biased views of oneself, of the world in general, and of the future) to reduce depression [53]. Table 1 presents an overview of modules. Therapist chat sessions were coscheduled by participant and therapist each week and were conducted inside the treatment platform. Sessions dealt with the previous and current module and focused on process-related aspects of treatment: identifying problems, examining the patient’s cognitions, encouragement, answering questions, and assisting with homework assignments. Participants who agreed were sent reminders before sessions, those who missed a session were offered a new time on the basis of their therapists’ remaining availability for the week. In addition to sessions, therapists responded to homework assignments and questions within 24 h on weekdays. The treatment was to be completed on a preset pace of 8 weeks, thereafter participants had access to the program for 4 additional weeks without therapist support.

Compared with our previous study [47], the treatment protocol was revised according to participants feedback: sessions were prolonged from 30 to 45 min, texts in some modules were revised to present more clearly (eg, reworked sentences, a more clear description of the rationale for exposure), and information procedures given to participants about weekly themes and goals were standardized. The overall conceptual model of delivery was kept intact.

Controls were assigned to a therapist and received an introductory personal platform in-mail from their therapist, informing them that there would be weekly assessments and that their assessments were to be viewed by their therapist to monitor their mental health state. They were informed that their therapist might contact them to follow-up on their wellbeing. While being in the control group, participants were allowed to seek regular care, which in Sweden is for free for adolescents.

**Table 1:** Internet-delivered cognitive behavior therapy intervention overview.

Week	Web-based session	Assignment/exercise
1	Psychoeducation	Write history, set goals
2	Analysis of behavior	Identify dysfunctional and functional schemas
3	Behavioral activation	Mood-activity diary
4	Behavioral activation	Mood-activity diary
5	Cognitive restructuring	Identify and challenge thoughts
6	Psychoeducation (anxiety)	Anxiety management, graded exposure
7	Emotional recognition	Coping strategies, self-esteem, affect regulation
8	Maintenance	Relapse prevention, treatment summary

### Therapists and Safety Procedures

In total, 6 CBT *therapists* in training conducted study assessments and treated 5 to 6 ICBT participants and monitored 5 to 6 control participants each. Before assessments, therapists received training in clinician interview (½ day), ICBT (written material), and how to navigate the treatment platform (½ day, on demand). Therapists received 60 min of clinical supervision on a weekly basis from clinical psychologists with expertise in adolescent psychopathology and delivery of ICBT. Communication and records (eg, chat, platform messages, and study consent) were available for the participant and therapist to view at any time. A short version of the Mood and Feelings Questionnaire (MFQ-13) and the suicidal ideation item from the Patient Health Questionnaire were used for weekly monitoring of depression. Participants were instructed to immediately contact their therapist in the event of feeling worse and were informed that their therapist might contact them in case of noncompletion. Scores and messages were monitored on a daily basis. In cases of suicidal ideation or significant deterioration, participants were immediately followed up by email and phone. The study collected participants personal identity number and address, and informed participants that in the event of imminent crisis, the study would break confidentiality to pursue appropriate follow-up.

### Outcomes

The primary outcome was self-reported depression severity at posttreatment, measured by the BDI-II [48]. Secondary outcomes included the MFQ [54]; the beck anxiety inventory (BAI) [55]; the social interaction anxiety scale (SIAS) [56]; the general self-efficacy scale (GSE) [57]; the credibility expectancy questionnaire [58]; the working alliance inventory (WAI-S) [59] and the Brunnsviken Brief Quality of life scale (BBQ) [60]; all self-report scales were completed over Web. The MINI was readministered over phone at posttreatment to assess depression diagnosis, assessors were not blinded to participant allocation at posttreatment.

### Analyses

Our previous study [47] was used as a reference for power calculations. To detect a similar effect size (Cohen *d*=0.70) at posttreatment, with a 2-tailed 5% significance level and a power of 80%, a total sample size of 72 was required. *A priori:* Participants were included in statistical analyses according to the intention-to-treat (ITT) principle. Missing data were handled using multiple imputations. Differences in primary outcomes were evaluated pre- to posttreatment by analysis of covariance (ANCOVA) using baseline values as covariate at the *P*<.05 level. Effect sizes were calculated based on imputed values and observed standard deviations. *Post hoc*: Independent *t* tests and Pearson chi-square tests were used to detect possible baseline differences between groups and percentage decrease in symptoms, respectively. Little missing completely at random test was performed to test the assumption of data missing at random, Levene test was performed to test the assumption of equal variance between groups. Completers were included in complementary statistical analyses for the primary and secondary outcomes. Clinically significant change [61] was determined by investigating the number of participants falling *2 SD* below the pretreatment mean for both conditions on the primary outcome, while fulfilling The reliable change index criteria [62], a psychometric criterion used to determine whether an individual change score between baseline and posttreatment assessment is significantly greater than a difference that could have occurred because of random measurement error. A criterion of 30% or more increase on the primary outcome from baseline to posttreatment was used to determine significant deterioration.

## Results

### Participant Flow

**Figure 1:**
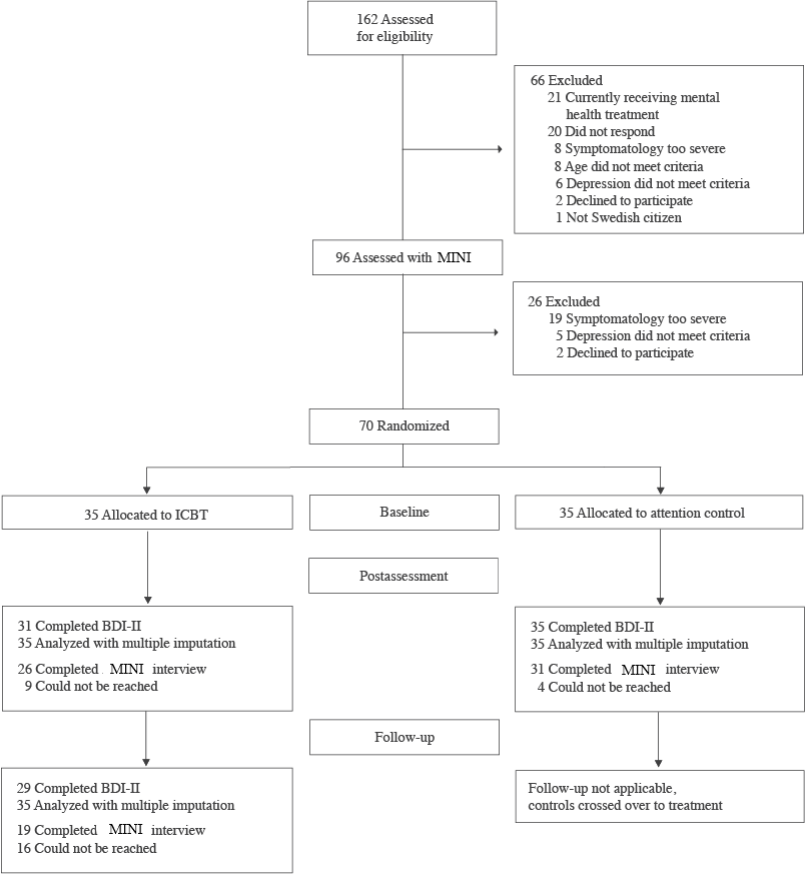
Participants’ flow through the study. MINI: Mini-International Neuropsychiatric Interview; ICBT: internet-delivered cognitive behavioral therapy; BDI-II: Beck Depression Inventory-II.

Figure 1 presents a Consolidated Standards of Reporting Trials diagram of the participant flow through the study. During 2 weeks of registration, 162 individuals completed the initial Web-based screening, out of whom, 12 identified as males. For included participants, the mean time from initial screening to the initiation of treatment or control was 12.7 days (range 4-17 days). No significant between-group differences were found at baseline regarding demographics or outcome measures. Table 2 presents the study sample baseline characteristics. During the treatment period, the average completion of the weekly assessment (both allocations) was 93% (range 84%-100%). Table 3 presents completion rates for primary and secondary outcomes at posttreatment. Post hoc analyses showed no differences between those lost to posttreatment and the rest of the sample on any outcome measures at baseline.

**Table 2:** Baseline characteristics of participants.

Characteristics		ICBT^a^ (n=35)	Control (n=35)
Female, n (%)		32 (91)	35 (100)
Age, mean (SD)		17.5 (1.1)	17.5 (1.2)
**Occupation, n (%)**			
	Studying full time	28 (80)	32 (91)
	Hiatus/dropout	2 (6)	0 (0)
	Working	5 (14)	3 (9)
**Residence, n (%)**			
	City	10 (29)	7 (20)
	Small town/country side	25 (71)	28 (80)
**Family, n (%)**			
	Two-parent household	17 (49)	9 (26)
	Other	18 (51)	26 (74)
	Parent(s) country of birth other than Sweden	7 (20)	7 (20)
**Major depressive episode^b^, n (%)**		27 (77)	26 (74)
	18-19 years	14 (74)	16 (80)
	15-17 years	13 (81)	10 (67)
**Comorbid anxiety diagnosis, n (%)**			
	Any	25 (71)	24 (69)
	Generalized anxiety disorder	14 (40)	15 (43)
	Social anxiety disorder	16 (46)	10 (29)
	Panic disorder	11 (31)	11 (31)
	Agoraphobia	4 (11)	7 (20)
Guardians informed about mental health state, n (%)		25 (71)	25 (71)
**Previous treatment history, n (%)**		10^c^ (29)	15^d^ (43)
	Psychotherapy treatment	10 (29)	14 (40)
	Psychotropic medication	1 (3)	4 (11)
**Current treatment, n (%)**		7^e^ (20)	3 (9)
	Counselor support	3 (9)	1 (3)
	Psychotropic medication	5 (14)	2 (6)

^a^ICBT: internet-delivered cognitive behavioral therapy.

^b^Confirmed in The Mini-International Neuropsychiatric Interview 7.0.

^c^One participant had experience of psychotherapy treatment as well as psychotropic treatment, thus total n=10.

^d^Some participants had experience of both types of treatment.

^e^Some participants had current experience of support as well as psychotropic medication.

**Table 3:** Participants’ assessment completion.

Measure completed	Posttreatment		12 months	
	ICBT^a^	Control	ICBT	Control
BDI-II^b^, n (%)	31 (89)	35 (100)	29 (83)	—^c^
BBQ^d^, n (%)	31 (89)	35 (100)	28 (80)	—
MFQ^e^, n (%)	31 (89)	34 (97)	28 (80)	—
BAI^f^, n (%)	31 (89)	34 (97)	28 (80)	—
SIAS^g^, n (%)	31 (89)	34 (97)	28 (80)	—
GSE^h^, n (%)	31 (89)	34 (97)	27 (77)	—

^a^ICBT: internet-delivered cognitive behavioral therapy.

^b^BDI-II: Beck Depression Inventory II.

^c^Data not applicable.

^d^BBQ: Brunnsviken Brief Quality of Life Inventory.

^e^MFQ: Mood and Feelings Questionnaire.

^f^BAI: Beck Anxiety Inventory.

^g^SIAS: Social Interaction Anxiety Scale.

^h^GSE: General Self-Efficacy scale.

### Primary Outcome

**Figure 2:**
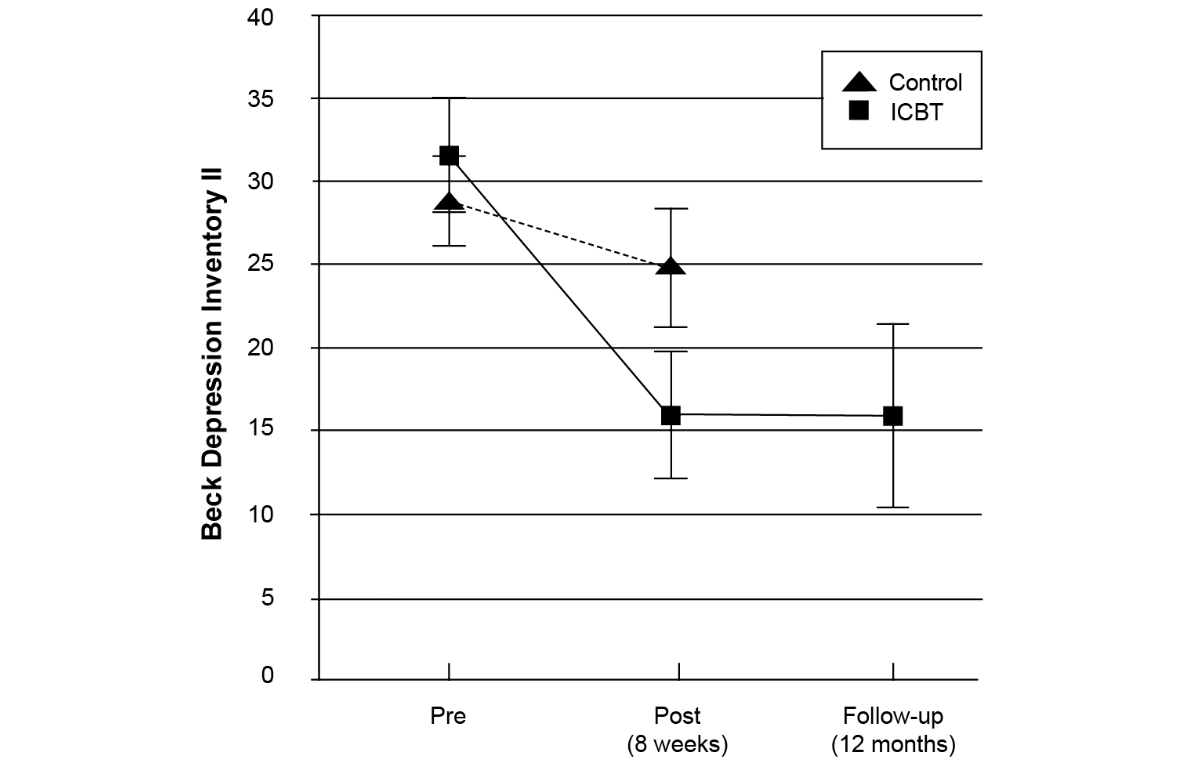
Change over time in depression severity (95% CIs) at baseline, posttreatment, and 12-month follow-up. ICBT: internet-delivered cognitive behavioral therapy.

Table 4 presents pre and posttreatment assessments including effect sizes, means, and standard deviations for both groups, at pre and posttreatment. For the primary outcome measure BDI-II, analyses with ANCOVA with baseline scores as covariate revealed a significant effect between groups (*F*_1,67_=22.23, *P*<.001) at posttreatment. The corresponding between-group effect size was *d*=0.86 (95% CI 0.37-1.35). A post hoc completer analysis (n=66) showed a similar result (*d*=0.99, 95% CI 0.48-1.51; *P*<.001). Figure 2 illustrates within-group improvements on the BDI-II. The ICBT group was reassessed 12 months following treatment. Analysis with paired *t* test from posttreatment to follow-up (ITT analysis) indicated that there was no difference in depression level from posttreatment assessment to the follow-up (BDI-II, *P*=.96).

**Table 4:** Means (SD) and effect sizes (Cohen *d*) with 95% CI for continuous outcome variables, with missing data imputed.

Measure		Baseline, mean (SD)	Posttest, mean (SD)	12 Months, mean (SD)	Cohen *d* between-group posttest (95% CI)
**BDI-II^a^**					
	ICBT^b^	31.6 (10.0)	16.0 (11.3)	15.9 (16.1)	0.86 (0.37 to 1.35)^c^
	Control	28.8 (7.9)	24.8 (10.4)	—^d^	—
**MFQ^e^**					
	ICBT	36.0 (10.7)	24.3 (12.8)	21.7 (17.4)	0.58 (0.10 to 1.06)^f^
	Control	35.2 (9.4)	31.0 (9.8)	—	—
**BBQ^g^**					
	ICBT	35.8 (18.1)	46.7 (21.3)	48.3 (27.0)	0.34 (0.19 to 1.15)^f^
	Control	38.7 (17.2)	39.1 (15.7)	—	—
**BAI^h^**					
	ICBT	28.6 (11.9)	16.6 (10.3)	15.8 (12.7)	0.30 (–0.17 to 0.77)
	Control	25.5 (11.2)	20.0 (9.3)	—	—
**SIAS^i^**					
	ICBT	45.2 (19.2)	35.4 (19.0)	37.4 (22.9)	0.05 (–0.41 to 0.52)
	Control	39.5 (16.4)	35.1 (14.3)	—	—
**GSE^j^**					
	ICBT	21.3 (5.6)	22.9 (7.5)	24.3 (9.6)	0.10 (–0.37 to 0.56)
	Control	22.2 (4.6)	23.0 (5.0)	—	—

^a^BDI-II: Beck Depression Inventory II.

^b^ICBT: internet-delivered cognitive behavioral therapy.

^c^*P*<.001.

^d^Data not applicable.

^e^MFQ: Mood and Feelings Questionnaire.

^f^*P*<.01.

^g^BBQ: Brunnsviken Brief Quality of Life Inventory.

^h^BAI: Beck Anxiety Inventory.

^i^SIAS: Social Interaction Anxiety Scale.

^j^GSE: General Self-Efficacy Scale.

### Response and Remission

We performed post hoc analyses to further investigate potential change in depression symptom level, with missing cases (ICBT n=4) categorized as not improved. At posttreatment, a higher proportion of ICBT participants (46%, 16/35) than controls (11%, 4/35) showed clinically significant improvement (χ^2^_1,70_=10.1; *P*=.001), defined as scoring *2 SD* below the pretreatment mean for both conditions on the BDI-II, while also fulfilling the reliable change index criteria [62]. In addition, the number of participants presenting a decrease of ≥30% in BDI-II score from baseline to posttreatment assessment, and BDI-II ≥13, and BDI-II ≥10 at postassessment was investigated as these measures have previously been used to define significant changes or cut-offs [48]. Significant differences between groups were found for all measures (*P*=.004, *P*=.004, *P*<.001). Participants were reassessed with the MINI at posttreatment. Of those participants that had fulfilled DSM-5 criteria for MDE at baseline (ICBT n=27; Control n=26), a higher proportion of ICBT participants (56%, 15/27) than controls (27%, 7/26) no longer met diagnostic criteria at posttreatment (χ^2^_1,53_=2.0; *P*=.03); missing cases were categorized as not improved (ICBT n=7; Control n=4).

### Secondary Outcomes

At posttreatment, a significant effect was found for the secondary depression outcome MFQ (*F*_1,67_=9.33; *P*=.003), corresponding to a medium between-group effect of *d*=0.58 (95% CI 0.10-1.06). A post hoc completer analysis (n=66) showed a similar result (*d*=0.67; 95% CI 0.18-1.17; *P*=.002). Analysis with paired *t* test from posttreatment to follow-up (ITT analysis) indicated that there was no difference in depression level from posttreatment assessment to the follow-up (MFQ, *P*=.25). The ANCOVA for posttreatment change in quality of life (BBQ) revealed a significant effect between groups (*F*_1,67_=8.73; *P*=.004). The ANCOVA for pre to posttreatment change in anxiety (BAI) showed no significant effect between groups (*F*_1,67_=3.95; *P*=.051), nor did the ANCOVAs for self-efficacy (GSE; *P*=.81) or social anxiety (SIAS, *P*=.86).

### Program Use

ICBT participants logged into the treatment platform for a mean of 28.4 times (SD 14.6) and completed on average 78% of available modules (mean 6.2 of 8 modules, SD 2.28), and on average 71% of available therapist sessions (mean 5.7 of 8 sessions, SD 2.67). The average total completion was 74% of all 16 available sessions and modules (11.8/16, SD 4.82). In total, 17% participants (6/35) completed less than half of the available treatment modules. No relationship was found between the number of completed sessions and modules and treatment outcome (*P*=.10). Therapists spent on average 43.6 min (SD 19.4) on each participant every week (if only including recordings that included therapist sessions, mean 55.8 min on each participant every week). ICBT participants’ ratings of treatment credibility (C-scale) showed an average rating of 18.5 (SD 4.17) out of a maximum total of 27 (highest credibility). The average score on items of therapeutic alliance (WAI-S) was 4.95 (SD 0.63) out of a maximum of 7 (highest satisfaction).

### Negative Outcomes

One participant in the ICBT group deteriorated significantly during the course of treatment and was directed to standard care services while being maintained in the study. Post hoc analyses showed that no participant deteriorated significantly following treatment, defined as an increase of 30% or more on the BDI-II from baseline to posttreatment. If those lost to posttreatment assessment were categorized as having deteriorated (n=4), the rate in the ICBT group would be 11%.

## Discussion

We examined whether ICBT blended with weekly synchronous therapist chat sessions was effective in reducing depression when compared with minimal attention control. The intervention was evaluated with adolescents 15 to 19 years of age (mean 17.5 years) suffering from depressive symptoms, including but not restricted to, MDE. Our report demonstrates the efficacy of the intervention and highlights the potential of a Web, text-based CBT approach to reach and treat adolescents suffering depression.

### Principal Findings

We found superiority for the intervention based on the primary outcome measure (BDI-II score at week 8), corresponding to a large between-group effect size (*d=*0.86). Significant effects on depression symptom level were also observed in the secondary self-reported depression outcome (MFQ, *d*=0.58), as well as in the number of cases in remission from a major depressive episode (determined using the MINI), further supporting that the intervention was effective. At the 12-month follow-up, depression levels in the treatment group were similar to those observed at posttreatment, follow-up data were not available for the control group. The results for the depression outcomes are in line with, and for the primary outcome surpass, the findings in our previous study that examined the intervention [47]. Although it is important to consider that we made changes to the protocol between studies—foremost, session time was prolonged—the overall conceptual model of delivery was kept intact, and we used comparable design, measures, and procedures across studies. We therefore consider that the findings in this study provide further support for the efficacy of ICBT modules blended with chat sessions to reduce adolescent depression.

### Relation to Previous Research

Our protocol included more therapist support than typically found in ICBT. We learned in our previous study that therapist sessions were utilized (completion, mean 78%) but perceived short, and in line with participants’ feedback, we prolonged session time in this study. Sessions were completed at a similar degree in this study (mean 71%) with fewer comments on sessions length. Possibly, the extension in time resulted in chat sessions being perceived as sufficient. Modules and sessions were completed at a similar degree, and few participants completed less than half of the treatment. This may explain why we found no association between dose and depression outcomes. Our tentative conclusion, based on participant feedback, program completion, and findings with the therapist work-alliance (WAI-S), is that real-time therapist interaction is indeed desired when offered in tandem with ICBT material, and that completely text-based sessions seem to generate a meaningful therapeutic relationship for young individuals. We treated participants with high depression severity, and it may be that an ICBT format with strong therapist support, *blended treatment,* can better address clinical levels of depression than highly automated interventions do, because blended treatment can be tailored to suit individual needs, help with motivation, and provide more opportunity to monitor signs of improvement and deterioration, respectively [37,63]. In this context, Sethi et al have previously reported a blended approach, consisting of Web-based ICBT and in-person sessions, to be superior to stand-alone Web-based ICBT, and similar to in-person CBT in reducing depression in young adults (mean 19.5/20.1 years [64,65]). Chat sessions in group format to deliver ICBT with adolescents and young adults (mean 20.9 years) has previously been evaluated in a study by van der Zanden et al [66]. They too reported a large effect size on depressive symptoms against waitlist at posttreatment (*d*=0.94), and similar rate of clinically reliable change (56%) as in this study.

On a general level, the completion rate in this study compares favorably with what is reported for Web-based interventions [67], including for youth [33], and it should be considered that we did not offer any payment for completion or assessment. The depression reduction observed in this study is moreover in line with meta-analyses suggesting that computer and Web-based ICBT for youth depression produces effect [11,32], and more broadly, that coached Web-based interventions fare better than self-guided [33]. Existing studies, however, vary considerable in relation to the populations, delivery mode, and measures in focus, making comparison difficult. For example, our obtained effect size is in line with the larger effect size found for computer-based and Web-based CBT with adolescents aged >13 years against waitlist (*g*=0.95), as compared with children (*g*=0.51), reported in a meta-analysis subanalysis [11], but the authors did not report interventions for anxiety and depression programs separately or differentiated on level of coach support. Most of previous RCT’s that have focused on elevated depression in adolescents and young adults [68-78] have evaluated ICBT self-guided programs [68-72]; telephone calls could occur [69], or interventions that are not ICBT models, but rather computerized programs (eg, fantasy games) with support or oversight at education site, school, or at home [73-78]. To the best of our knowledge, no previous ICBT programs have involved individual therapist sessions with adolescents with current major depressive episode. Although our results are promising and could be associated with the level of therapist support, this was not established by the methods used, and we cannot rule out that other factors have played a role, for example, time to treatment, and initial depression severity. This should be addressed in future studies.

### Clinical Implications

This study focused on efficacy, nevertheless findings may in several aspects have clinical implication. First, we used a recruitment strategy that is similar to the way in which some young individuals enroll for Web-based mental health intervention [79], thus giving ecological validity to this study and the population with which it is conducted. Second, our findings support that reducing adolescent depression, including major depressive episode, is possible using a genuinely Web-based CBT approach. Third, our results compare favorably with those reported in meta-analyses on in-person CBT with youth depression as compared with active and nonactive control conditions [80-82], and more broadly, with effects for in-person psychotherapies for youth depression (*g*=0.29) [83]. Fourth, observed diagnosis remission rates are in the lower range compared with studies that have reported remission for in-person CBT: 61% to 87% [84-86]. Participants in our study had high depression scores initially, with large reductions needed to reach the criteria for remission. It has been suggested that young people who access mental health resources over Web are likely to be in greater distress in comparison with those accessing in-person services (given more rapid access to Web, they are closer in time to their symptoms [87]), which point toward the relevance of Web-based behavioral interventions extending to address clinical depression. Fifth, there are concerns among stakeholders about the safety and effectiveness of internet interventions for depression, particularly regarding offering such interventions to youth [88,89], which is why this study reports on potential negative effects related to intervention. We found 1 participant deteriorating significantly during the course of treatment. In comparison, it has been reported that 14% to 24% of young people receiving psychotherapy in US outpatient mental health services experience significant deterioration following treatment [90]. Concerning ICBT, a review reports 5.8% of adult study participants experiencing significant deterioration following self-guided programs [91]; no review has investigated youth. Sixth, in contrast to clinical practice [92] and research in many places, this study did not require participants to visit a care facility or school counselor or inform their guardians to receive treatment. This was to acknowledge that some adolescents do not want to, or cannot involve, their parents in their difficulties [93], and that this reluctance may delay or impede evaluation and treatment [94]. We found the intervention to attract young individuals (mean 17.5 years), some reporting that they had not informed parent(s) about their mental state (29%). The findings on participants’ characteristics, as well as the other findings reported on here, are very similar to those in our previous study [47], and further contribute to reveal who may be reached and treated when similar interventions are offered. If the aim is to treat young individuals, at an early stage of the disorder, the conditions required to receive treatment may benefit from becoming more inclusive.

### Strengths and Limitations

Strengths of the study include a rigorous design, use of a primary outcome with strong psychometric evidence, and adequate power with regards to detecting between-group differences in the included depression outcomes. In relation to the reproducibility challenges in psychology, it is positive that our results are in line with those in our previous studies on ICBT, although independent evaluations are necessary. We consider it a strength that the intervention was investigated with adolescents 15 to 19 years of age as opposed to a wider age range. It is indicated that the causes and constructs of depression may differ between children and adolescents [3], thus studies including both groups could possibly suffer from these differences interacting and interfering with treatment outcomes. A number of limitations should be considered: this study is one of the first on chat-supported ICBT, thus we believed that no evidence-based treatment control group would be appropriate. Nevertheless, it is a limitation that an active control intervention was not included. In line with discussions [21] regarding what is considered an intervention, we moreover labeled our control condition an attention control, as controls were thoroughly assessed and monitored and interacted with. The appropriateness of the label can be discussed. Treatment was open label and participants’ awareness of their allocation may have affected self-reported outcomes; similarly, the clinical interviews were not blinded at posttreatment, which calls for caution when interpreting remission rates. The reduction on anxiety ratings cannot be explained by the treatment as the control group also improved. As the intervention focused on depression, it is possible that it was not effective enough to result in major reductions in anxiety in the treatment group. Participants in the study were almost entirely female, so results cannot be generalized to males. Depression is more prevalent among women but gender distributions for Web-based interventions and support can be even more skewed [87,95-96]. There are differences in internet use patterns and preferences between adolescent girls and boys [43], and possibly these differences interact with Web-based interventions as they currently are offered. It has been discussed that young boys are more likely than girls to seek help as a consequence of being influenced by others, and this could explain their relatively low enrollment in Web-based interventions, given that such enrollment is often more dependent on self-motivation and in many cases self-referral [87]. Our study recruited via social media, and moreover via an account focusing on coping with mental health issues; this may have influenced gender uptake as well as attracted particularly motivated participants. Although ours and previous findings point to the need of alterations in recruitment and design so that similar Web-based intervention can reach boys, the positive findings with girls should not be undervalued and the intervention should be regarded complementary among others. Not limiting to gender, focused (tailored) approaches rather than broad and universal interventions may be more relevant to desired target groups, and thus possibly more successful. For example, using a narrow age span, in our experience, facilitates the creation of content that is developmentally appropriate. Finally, while our positive findings may relate to the novel features of the intervention, the study contributes limited information on how the effects were achieved, for example, on the specific impact of therapist support. Lacking information on what factors influence outcomes in ICBT with youth is a common study limitation [34].

### Future Research

The next step is to extend focus to uncovering the effect of therapist interaction, and other theoretical and contextual treatment components in ICBT, to contribute to our understanding of what components positively affect treatment outcomes in psychotherapy with youth depression and how they do so. Multiphase optimization strategies that include factorial experimental designs pose as viable options [97]. Technology-based treatment enables new evaluation strategies and the current approach in particular produces vast amounts of data from patient-therapist correspondences that benefit from natural language processing to help understand and refine conversation [98,99]. Qualitative investigation will help to ensure that components perceived essential by young individuals are not lost but enhanced in further developments.

### Conclusions

This study investigated treatment consisting of Web-based CBT self-help material and weekly therapist chat sessions, *blended treatment,* for adolescent depression. The intervention attracted adolescents in need of mental health assistance and demonstrated positive completion rates in combination with substantial improvement in depression symptoms. The results are similar to those in our previous study, further demonstrating the potential of a text-based blended model to deliver CBT in accordance with the urgent need for accessible behavioral intervention for youth.

## Appendix

Multimedia Appendix 1 CONSORT-EHEALTH checklist (V 1.6.1).

## Abbreviations

ANCOVA: analysis of covariance
BAI: Beck Anxiety Inventory
BBQ: Brunnsviken Brief Quality of Life Scale
BDI-II: Beck Depression Inventory II
CBT: cognitive behavioral therapy
DSM-5: Diagnostic and Statistical Manual of Mental Disorder criteria
GSE: General Self-Efficacy Scale
ICBT: internet-delivered cognitive behavior therapy
ITT: intention-to-treat
MFQ: Mood and Feelings Questionnaire
MINI: Mini-International Neuropsychiatric Interview
SIAS: Social Interaction Anxiety Scale
WAI-S: Working Alliance Inventory
