# Supplementary material for: Evaluating the Efficacy of Internet-Delivered Cognitive Behavioral Therapy Blended With Synchronous Chat Sessions to Treat Adolescent Depression: Randomized Controlled Trial
Source: J Med Internet Res. 2019 Nov 1;21(11):e13393. doi: 10.2196/13393 (PMC6858617; doi:10.2196/13393)
Supplement: Multimedia Appendix 1 [file jmir_v21i11e13393_app1.pdf]

CONSORT-EHEALTH (V 1.6.1) - Submission/Publication Form

The CONSORT-EHEALTH checklist is intended for authors of randomized trials evaluating web-based and Internet-based applications/interventions, including mobile interventions, electronic games (incl multiplayer games), social media, certain telehealth applications, and other interactive and/or networked electronic applications. Some of the items (e.g. all subitems under item 5 - description of the intervention) may also be applicable for other study designs.

The goal of the CONSORT EHEALTH checklist and guideline is to be
a) a guide for reporting for authors of RCTs,
b) to form a basis for appraisal of an ehealth trial (in terms of validity)

CONSORT-EHEALTH items/subitems are MANDATORY reporting items for studies published in the Journal of Medical Internet Research and other journals / scientific societies endorsing the checklist.

Items numbered 1., 2., 3., 4a., 4b etc are original CONSORT or CONSORT-NPT (non-pharmacologic treatment) items.
Items with Roman numerals (i., ii, iii, iv etc.) are CONSORT-EHEALTH extensions/clarifications.

As the CONSORT-EHEALTH checklist is still considered in a formative stage, we would ask that you also RATE ON A SCALE OF 1-5 how important/useful you feel each item is FOR THE PURPOSE OF THE CHECKLIST and reporting guideline (optional).

Mandatory reporting items are marked with a red *.
In the textboxes, either copy & paste the relevant sections from your manuscript into this form - please include any quotes from your manuscript in QUOTATION MARKS,
or answer directly by providing additional information not in the manuscript, or elaborating on why the item was not relevant for this study.

YOUR ANSWERS WILL BE PUBLISHED AS A SUPPLEMENTARY FILE TO YOUR PUBLICATION IN JMIR AND ARE CONSIDERED PART OF YOUR PUBLICATION (IF ACCEPTED).
Please fill in these questions diligently. Information will not be copyedited, so please use proper spelling and grammar, use correct capitalization, and avoid abbreviations.

DO NOT FORGET TO SAVE AS PDF _AND_ CLICK THE SUBMIT BUTTON SO YOUR ANSWERS ARE IN OUR DATABASE !!!

Citation Suggestion (if you append the pdf as Appendix we suggest to cite this paper in the caption):
Eysenbach G, CONSORT-EHEALTH Group
CONSORT-EHEALTH: Improving and Standardizing Evaluation Reports of Web-based and Mobile Health Interventions
J Med Internet Res 2011;13(4):e126
URL: http://www.jmir.org/2011/4/e126/
doi: 10.2196/jmir.1923
PMID: 22209829

* Required

Your name *

First Last

Naira Topooco

Primary Affiliation (short), City, Country *

University of Toronto, Toronto, Canada

Linköping University, Sweden

Your e-mail address *

abc@gmail.com

naira.topooco@liu.se

Title of your manuscript *

Provide the (draft) title of your manuscript.

Evaluating the Efficacy of Internet-Delivered Cognitive Behavioral Therapy Blended With Synchronous Chat Sessions to Treat Adolescent Depression: Randomized Controlled Trial

Name of your App/Software/Intervention *

If there is a short and a long/alternate name, write the short name first and add the long name in brackets.

Blended treatment; ICBT blended with weekly r

Evaluated Version (if any)

e.g. "V1", "Release 2017-03-01", "Version 2.0.27913"

Your answer

Language(s) *

What language is the intervention/app in? If multiple languages are available, separate by comma (e.g. "English, French")

Swedish

URL of your Intervention Website or App

e.g. a direct link to the mobile app on app in appstore (itunes, Google Play), or URL of the website. If the intervention is a DVD or hardware, you can also link to an Amazon page.

Your answer

URL of an image/screenshot (optional)

Your answer

Accessibility *

Can an enduser access the intervention presently?

- ○ access is free and open
- ◉ access only for special usergroups, not open
- ○ access is open to everyone, but requires payment/subscription/in-app purchases
- ○ app/intervention no longer accessible
- ○ Other:

Primary Medical Indication/Disease/Condition *

e.g. "Stress", "Diabetes", or define the target group in brackets after the condition, e.g. "Autism (Parents of children with)", "Alzheimers (Informal Caregivers of)"

Depression

Primary Outcomes measured in trial *

comma-separated list of primary outcomes reported in the trial

BDI-II; Beck depression Inventory II

Secondary/other outcomes

Are there any other outcomes the intervention is expected to affect?

MFQ; Mood and Feelings Questionnaire

Recommended "Dose" *

What do the instructions for users say on how often the app should be used?

- (x) Approximately Daily
- ( ) Approximately Weekly
- ( ) Approximately Monthly
- ( ) Approximately Yearly
- ( ) "as needed"
- ( ) Other:

Approx. Percentage of Users (starters) still using the app as recommended after 3 months *

- (x) unknown / not evaluated
- ( ) 0-10%
- ( ) 11-20%
- ( ) 21-30%
- ( ) 31-40%
- ( ) 41-50%
- ( ) 51-60%
- ( ) 61-70%
- ( ) 71%-80%
- ( ) 81-90%
- ( ) 91-100%
- ( ) Other:

Overall, was the app/intervention effective? *

- (x) yes: all primary outcomes were significantly better in intervention group vs control
- ( ) partly: SOME primary outcomes were significantly better in intervention group vs control
- ( ) no statistically significant difference between control and intervention
- ( ) potentially harmful: control was significantly better than intervention in one or more outcomes
- ( ) inconclusive: more research is needed
- ( ) Other:

Article Preparation Status/Stage *

At which stage in your article preparation are you currently (at the time you fill in this form)

- ○ not submitted yet - in early draft status
- ○ not submitted yet - in late draft status, just before submission
- ○ submitted to a journal but not reviewed yet
- ○ submitted to a journal and after receiving initial reviewer comments
- ◉ submitted to a journal and accepted, but not published yet
- ○ published
- ○ Other:

Journal *

If you already know where you will submit this paper (or if it is already submitted), please provide the journal name (if it is not JMIR, provide the journal name under "other")

- ○ not submitted yet / unclear where I will submit this
- ◉ Journal of Medical Internet Research (JMIR)
- ○ JMIR mHealth and UHealth
- ○ JMIR Serious Games
- ○ JMIR Mental Health
- ○ JMIR Public Health
- ○ JMIR Formative Research
- ○ Other JMIR sister journal
- ○ Other:

Is this a full powered effectiveness trial or a pilot/feasibility trial? *

- ○ Pilot/feasibility
- ◉ Fully powered

Manuscript tracking number *

If this is a JMIR submission, please provide the manuscript tracking number under "other" (The ms tracking number can be found in the submission acknowledgement email, or when you login as author in JMIR. If the paper is already published in JMIR, then the ms tracking number is the four-digit number at the end of the DOI, to be found at the bottom of each published article in JMIR)

- ○ no ms number (yet) / not (yet) submitted to / published in JMIR
- ● Other: 13393

TITLE AND ABSTRACT

1a) TITLE: Identification as a randomized trial in the title

1a) Does your paper address CONSORT item 1a? *

I.e does the title contain the phrase "Randomized Controlled Trial"? (if not, explain the reason under "other")

- ● yes
- ○ Other:

1a-i) Identify the mode of delivery in the title

Identify the mode of delivery. Preferably use “web-based” and/or “mobile” and/or “electronic game” in the title. Avoid ambiguous terms like “online”, “virtual”, “interactive”. Use “Internet-based” only if Intervention includes non-web-based Internet components (e.g. email), use “computer-based” or “electronic” only if offline products are used. Use “virtual” only in the context of “virtual reality” (3-D worlds). Use “online” only in the context of “online support groups”. Complement or substitute product names with broader terms for the class of products (such as “mobile” or “smart phone” instead of “iphone”), especially if the application runs on different platforms.

	1	2	3	4	5	
subitem not at all important	○	○	○	○	●	essential

Does your paper address subitem 1a-i? *

Copy and paste relevant sections from manuscript title (include quotes in quotation marks "like this" to indicate direct quotes from your manuscript), or elaborate on this item by providing additional information not in the ms, or briefly explain why the item is not applicable/relevant for your study

"Internet-delivered cognitive behavioral therapy blended with weekly real-time therapist sessions via chat; blended treatment"

1a-ii) Non-web-based components or important co-interventions in title

Mention non-web-based components or important co-interventions in title, if any (e.g., “with telephone support”).

	1	2	3	4	5	
subitem not at all important	○	○	○	○	◉	essential

Does your paper address subitem 1a-ii?

Copy and paste relevant sections from manuscript title (include quotes in quotation marks "like this" to indicate direct quotes from your manuscript), or elaborate on this item by providing additional information not in the ms, or briefly explain why the item is not applicable/relevant for your study

"Internet-delivered cognitive behavioral therapy blended with weekly real-time therapist sessions via chat; blended treatment"

1a-iii) Primary condition or target group in the title

Mention primary condition or target group in the title, if any (e.g., “for children with Type I Diabetes”)
Example: A Web-based and Mobile Intervention with Telephone Support for Children with Type I Diabetes: Randomized Controlled Trial

	1	2	3	4	5	
subitem not at all important	○	○	○	○	◉	essential

Does your paper address subitem 1a-iii? *

Copy and paste relevant sections from manuscript title (include quotes in quotation marks "like this" to indicate direct quotes from your manuscript), or elaborate on this item by providing additional information not in the ms, or briefly explain why the item is not applicable/relevant for your study

Adolescent Depression

1b) ABSTRACT: Structured summary of trial design, methods, results, and conclusions

NPT extension: Description of experimental treatment, comparator, care providers, centers, and blinding status.

1b-i) Key features/functionalities/components of the intervention and comparator in the METHODS section of the ABSTRACT

Mention key features/functionalities/components of the intervention and comparator in the abstract. If possible, also mention theories and principles used for designing the site. Keep in mind the needs of systematic reviewers and indexers by including important synonyms. (Note: Only report in the abstract what the main paper is reporting. If this information is missing from the main body of text, consider adding it)

	1	2	3	4	5	
subitem not at all important	○	○	○	◉	○	essential

Does your paper address subitem 1b-i? *

Copy and paste relevant sections from the manuscript abstract (include quotes in quotation marks "like this" to indicate direct quotes from your manuscript), or elaborate on this item by providing additional information not in the ms, or briefly explain why the item is not applicable/relevant for your study

"ICBT protocol blended with weekly real-time therapist sessions via chat; blended treatment, for adolescent depression, including major depressive episode (MDE)."; "allocated to either 8 weeks of treatment or to minimal attention control"

1b-ii) Level of human involvement in the METHODS section of the ABSTRACT

Clarify the level of human involvement in the abstract, e.g., use phrases like “fully automated” vs. “therapist/nurse/care provider/physician-assisted” (mention number and expertise of providers involved, if any). (Note: Only report in the abstract what the main paper is reporting. If this information is missing from the main body of text, consider adding it)

	1	2	3	4	5	
subitem not at all important	○	○	○	○	◉	essential

Does your paper address subitem 1b-ii?

Copy and paste relevant sections from the manuscript abstract (include quotes in quotation marks "like this" to indicate direct quotes from your manuscript), or elaborate on this item by providing additional information not in the ms, or briefly explain why the item is not applicable/relevant for your study

weekly real-time therapist sessions via chat

1b-iii) Open vs. closed, web-based (self-assessment) vs. face-to-face assessments in the METHODS section of the ABSTRACT

Mention how participants were recruited (online vs. offline), e.g., from an open access website or from a clinic or a closed online user group (closed usergroup trial), and clarify if this was a purely web-based trial, or there were face-to-face components (as part of the intervention or for assessment). Clearly say if outcomes were self-assessed through questionnaires (as common in web-based trials). Note: In traditional offline trials, an open trial (open-label trial) is a type of clinical trial in which both the researchers and participants know which treatment is being administered. To avoid confusion, use “blinded” or “unblinded” to indicated the level of blinding instead of “open”, as “open” in web-based trials usually refers to “open access” (i.e. participants can self-enrol). (Note: Only report in the abstract what the main paper is reporting. If this information is missing from the main body of text, consider adding it)

	1	2	3	4	5	
subitem not at all important	○	○	○	○	◉	essential

Does your paper address subitem 1b-iii?

Copy and paste relevant sections from the manuscript abstract (include quotes in quotation marks "like this" to indicate direct quotes from your manuscript), or elaborate on this item by providing additional information not in the ms, or briefly explain why the item is not applicable/relevant for your study

"ecruited through a community setting at the national level in Sweden"; "The primary outcome was self-reported depression level as measured with the Beck Depression Inventory II at posttreatment."

1b-iv) RESULTS section in abstract must contain use data

Report number of participants enrolled/assessed in each group, the use/uptake of the intervention (e.g., attrition/adherence metrics, use over time, number of logins etc.), in addition to primary/secondary outcomes. (Note: Only report in the abstract what the main paper is reporting. If this information is missing from the main body of text, consider adding it)

	1	2	3	4	5	
subitem not at all important	○	○	○	○	◉	essential

Does your paper address subitem 1b-iv?

Copy and paste relevant sections from the manuscript abstract (include quotes in quotation marks "like this" to indicate direct quotes from your manuscript), or elaborate on this item by providing additional information not in the ms, or briefly explain why the item is not applicable/relevant for your study

The average intervention completion was 74% (11.8 of 16 modules and sessions).

1b-v) CONCLUSIONS/DISCUSSION in abstract for negative trials

Conclusions/Discussions in abstract for negative trials: Discuss the primary outcome - if the trial is negative (primary outcome not changed), and the intervention was not used, discuss whether negative results are attributable to lack of uptake and discuss reasons. (Note: Only report in the abstract what the main paper is reporting. If this information is missing from the main body of text, consider adding it)

	1	2	3	4	5	
subitem not at all important	○	○	○	○	◉	essential

Does your paper address subitem 1b-v?

Copy and paste relevant sections from the manuscript abstract (include quotes in quotation marks "like this" to indicate direct quotes from your manuscript), or elaborate on this item by providing additional information not in the ms, or briefly explain why the item is not applicable/relevant for your study

-

INTRODUCTION

2a) In INTRODUCTION: Scientific background and explanation of rationale

2a-i) Problem and the type of system/solution

Describe the problem and the type of system/solution that is object of the study: intended as stand-alone intervention vs. incorporated in broader health care program? Intended for a particular patient population? Goals of the intervention, e.g., being more cost-effective to other interventions, replace or complement other solutions? (Note: Details about the intervention are provided in “Methods” under 5)

	1	2	3	4	5	
subitem not at all important	○	○	○	◉	○	essential

Does your paper address subitem 2a-i? *

Copy and paste relevant sections from the manuscript (include quotes in quotation marks "like this" to indicate direct quotes from your manuscript), or elaborate on this item by providing additional information not in the ms, or briefly explain why the item is not applicable/relevant for your study

Patient population/problem:
"Unipolar depressive disorders are the leading cause of disability-adjusted life years among adolescents aged 15 to 19 years globally [1].Despite the high level of disability, only a small fraction of young people in need have access to any kind of intervention [10,11]."

Goals of intervention:
"Worldwide, adaptions of in-person psychotherapy protocols into Web-based formats, the majority involving CBT, are emerging to bring mental health interventions to individuals who for different reasons are not reached by regular services. To include a strong therapist interaction in the form of sessions is in line with aggregated findings that support is desired and boosts the effect of ICBT. The inclusion of strong therapist interaction could moreover extend ICBT to appropriately address clinical depression in youth. In light of full-threshold depressive episodes being commonly experienced in adolescence and young adulthood despite prevention effort [46], this is highly relevant. This study investigated a treatment protocol consisting of ICBT modules blended with weekly therapist chat sessions for adolescent depression, including major depressive episode (MDE). "

2a-ii) Scientific background, rationale: What is known about the (type of) system

Scientific background, rationale: What is known about the (type of) system that is the object of the study (be sure to discuss the use of similar systems for other conditions/diagnoses, if appropiate), motivation for the study, i.e. what are the reasons for and what is the context for this specific study, from which stakeholder viewpoint is the study performed, potential impact of findings [2]. Briefly justify the choice of the comparator.

	1	2	3	4	5	
subitem not at all important	○	○	○	●	○	essential

Does your paper address subitem 2a-ii? *

Copy and paste relevant sections from the manuscript (include quotes in quotation marks "like this" to indicate direct quotes from your manuscript), or elaborate on this item by providing additional information not in the ms, or briefly explain why the item is not applicable/relevant for your study

"To include a strong therapist interaction in the form of sessions is in line with aggregated findings that support is desired and boosts the effect of ICBT. To include strong therapist support in the form of sessions (ie, blended treatment), and moreover, doing so with real-time texting is consistent with adolescents' media use preferences, while maintaining the combination of easy access and discretion offered in ICBT, which for young people may prove especially effective in overcoming barriers to behavioral intervention. The inclusion of strong therapist interaction could moreover extend ICBT to appropriately address clinical depression in youth. In light of full-threshold depressive episodes being commonly experienced in adolescence and young adulthood despite prevention effort [46], this is highly relevant.This study investigated a treatment protocol consisting of ICBT modules blended with weekly therapist chat sessions for adolescent depression, including major depressive episode (MDE). "

2b) In INTRODUCTION: Specific objectives or hypotheses

Does your paper address CONSORT subitem 2b? *

Copy and paste relevant sections from the manuscript (include quotes in quotation marks "like this" to indicate direct quotes from your manuscript), or elaborate on this item by providing additional information not in the ms, or briefly explain why the item is not applicable/relevant for your study

"We hypothesized that the intervention would outperform the control condition in reducing depression, corresponding to at least moderate between-group effect."

METHODS

3a) Description of trial design (such as parallel, factorial) including allocation ratio

Does your paper address CONSORT subitem 3a? *

Copy and paste relevant sections from the manuscript (include quotes in quotation marks "like this" to indicate direct quotes from your manuscript), or elaborate on this item by providing additional information not in the ms, or briefly explain why the item is not applicable/relevant for your study

"In a 2-arm randomized controlled trial, adolescents were recruited in a community setting at the national level in Sweden and randomized (1:1 ratio) to ICBT (n=35) or to minimal attention control (n=35)".

3b) Important changes to methods after trial commencement (such as eligibility criteria), with reasons

Does your paper address CONSORT subitem 3b? *

Copy and paste relevant sections from the manuscript (include quotes in quotation marks "like this" to indicate direct quotes from your manuscript), or elaborate on this item by providing additional information not in the ms, or briefly explain why the item is not applicable/relevant for your study

There were no important changes to methods after trial commencement.

3b-i) Bug fixes, Downtimes, Content Changes

Bug fixes, Downtimes, Content Changes: ehealth systems are often dynamic systems. A description of changes to methods therefore also includes important changes made on the intervention or comparator during the trial (e.g., major bug fixes or changes in the functionality or content) (5-iii) and other “unexpected events” that may have influenced study design such as staff changes, system failures/downtimes, etc. [2].

	1	2	3	4	5	
subitem not at all important	○	○	○	◉	○	essential

Does your paper address subitem 3b-i?

Copy and paste relevant sections from the manuscript (include quotes in quotation marks "like this" to indicate direct quotes from your manuscript), or elaborate on this item by providing additional information not in the ms, or briefly explain why the item is not applicable/relevant for your study

Your answer

4a) Eligibility criteria for participants

Does your paper address CONSORT subitem 4a? *

Copy and paste relevant sections from the manuscript (include quotes in quotation marks "like this" to indicate direct quotes from your manuscript), or elaborate on this item by providing additional information not in the ms, or briefly explain why the item is not applicable/relevant for your study

"Eligible participants were 15 to 19 years, suffered depressive symptoms (≥14 points on the Beck Depression Inventory II; BDI-II [48]), and presented at least 4 symptoms including 1 core symptom, or fulfilled criteria for major depressive episode (MDE) according to The Mini-International Neuropsychiatric Interview (MINI 7.0 [49]). We excluded individuals who were receiving psychological therapy, were alcohol or drug dependent, showed severe suicidal ideation, or who had severe comorbid psychiatric conditions (eg, bipolar disorder or psychotic symptoms). Comorbid anxiety disorder(s) were allowed if depression was the principle concern. Medication for anxiety, depression, or Attention deficit hyperactivity disorder was accepted if the dose had been stable >1 month before the study. Minors aged 15 to 17 years were included in line with Swedish research legislation,"

4a-i) Computer / Internet literacy

Computer / Internet literacy is often an implicit “de facto” eligibility criterion - this should be explicitly clarified.

	1	2	3	4	5	
subitem not at all important	○	○	○	◉	○	essential

Does your paper address subitem 4a-i?

Copy and paste relevant sections from the manuscript (include quotes in quotation marks "like this" to indicate direct quotes from your manuscript), or elaborate on this item by providing additional information not in the ms, or briefly explain why the item is not applicable/relevant for your study

4a-ii) Open vs. closed, web-based vs. face-to-face assessments:

Open vs. closed, web-based vs. face-to-face assessments: Mention how participants were recruited (online vs. offline), e.g., from an open access website or from a clinic, and clarify if this was a purely web-based trial, or there were face-to-face components (as part of the intervention or for assessment), i.e., to what degree got the study team to know the participant. In online-only trials, clarify if participants were quasi-anonymous and whether having multiple identities was possible or whether technical or logistical measures (e.g., cookies, email confirmation, phone calls) were used to detect/prevent these.

	1	2	3	4	5	
subitem not at all important	○	○	○	○	◉	essential

Does your paper address subitem 4a-ii? *

Copy and paste relevant sections from the manuscript (include quotes in quotation marks "like this" to indicate direct quotes from your manuscript), or elaborate on this item by providing additional information not in the ms, or briefly explain why the item is not applicable/relevant for your study

The intervention was purely web-based. Assessments were conducted in the form of web-based sel-report, and phone interviews (MINI 7.0).

"Participants were recruited by social media posts from study staff including one guest post in a wide-reaching Instagram account focusing on coping with mental health issues. Posts described the opportunity to receive Web-based psychological treatment within a research study. otential participants were directed to the study website and registered for the study by creating a user account, providing informed consent (checking a box), and completing a Web-based screen (demographics and self-reported outcome measures). An encrypted Web-based treatment platform, Iterapi, was used to collect screening data [50]. Individuals who showed initial eligibility were invited to a phone interview with study staff to confirm eligibility using the full MINI, to determine matureness to participate, to obtain verbal consent, and to confirm identity (name, address, and personal identity number). Before the randomization, eligible participants were requested to sign a digital consent sheet (full name with digital date stamp) to confirm their willingness to participate in the study."

4a-iii) Information giving during recruitment

Information given during recruitment. Specify how participants were briefed for recruitment and in the informed consent procedures (e.g., publish the informed consent documentation as appendix, see also item X26), as this information may have an effect on user self-selection, user expectation and may also bias results.

	1	2	3	4	5	
subitem not at all important	○	○	○	●	○	essential

Does your paper address subitem 4a-iii?

Copy and paste relevant sections from the manuscript (include quotes in quotation marks "like this" to indicate direct quotes from your manuscript), or elaborate on this item by providing additional information not in the ms, or briefly explain why the item is not applicable/relevant for your study

"Participants were recruited by social media posts from study staff including one guest post in a wide-reaching Instagram account focusing on coping with mental health issues. Posts described the opportunity to receive Web-based psychological treatment within a research study."

4b) Settings and locations where the data were collected

Does your paper address CONSORT subitem 4b? *

Copy and paste relevant sections from the manuscript (include quotes in quotation marks "like this" to indicate direct quotes from your manuscript), or elaborate on this item by providing additional information not in the ms, or briefly explain why the item is not applicable/relevant for your study

"Potential participants were directed to the study website and registered for the study by creating a user account, providing informed consent (checking a box), and completing a Web-based screen (demographics and self-reported outcome measures). An encrypted Web-based treatment platform, Iterapi, was used to collect screening data [50].
Individuals who showed initial eligibility were invited to a phone interview with study staff to confirm eligibility using the full MINI, to determine matureness to participate, to obtain verbal consent, and to confirm identity (name, address, and personal identity number)."

4b-i) Report if outcomes were (self-)assessed through online questionnaires

Clearly report if outcomes were (self-)assessed through online questionnaires (as common in web-based trials) or otherwise.

	1	2	3	4	5	
subitem not at all important	○	○	○	○	◉	essential

Does your paper address subitem 4b-i? *

Copy and paste relevant sections from the manuscript (include quotes in quotation marks "like this" to indicate direct quotes from your manuscript), or elaborate on this item by providing additional information not in the ms, or briefly explain why the item is not applicable/relevant for your study

"Potential participants were directed to the study website and registered for the study by creating a user account, providing informed consent (checking a box), and completing a Web-based screen (demographics and self-reported outcome measures). An encrypted Web-based treatment platform, Iterapi, was used to collect screening data [50].
The primary outcome was self-reported depression severity at posttreatment, measured by the BDI-II [48]"

4b-ii) Report how institutional affiliations are displayed

Report how institutional affiliations are displayed to potential participants [on ehealth media], as affiliations with prestigious hospitals or universities may affect volunteer rates, use, and reactions with regards to an intervention.(Not a required item – describe only if this may bias results)

	1	2	3	4	5	
subitem not at all important	○	◉	○	○	○	essential

Does your paper address subitem 4b-ii?

Copy and paste relevant sections from the manuscript (include quotes in quotation marks "like this" to indicate direct quotes from your manuscript), or elaborate on this item by providing additional information not in the ms, or briefly explain why the item is not applicable/relevant for your study

University logo (Linköping University) presented on study website

5) The interventions for each group with sufficient details to allow replication, including how and when they were actually administered

5-i) Mention names, credential, affiliations of the developers, sponsors, and owners

Mention names, credential, affiliations of the developers, sponsors, and owners [6] (if authors/evaluators are owners or developer of the software, this needs to be declared in a “Conflict of interest” section or mentioned elsewhere in the manuscript).

	1	2	3	4	5	
subitem not at all important	○	○	○	◉	○	essential

Does your paper address subitem 5-i?

Copy and paste relevant sections from the manuscript (include quotes in quotation marks "like this" to indicate direct quotes from your manuscript), or elaborate on this item by providing additional information not in the ms, or briefly explain why the item is not applicable/relevant for your study

Conflicts of Interest
None declared.

5-ii) Describe the history/development process

Describe the history/development process of the application and previous formative evaluations (e.g., focus groups, usability testing), as these will have an impact on adoption/use rates and help with interpreting results.

	1	2	3	4	5	
subitem not at all important	○	○	○	○	◉	essential

Does your paper address subitem 5-ii?

Copy and paste relevant sections from the manuscript (include quotes in quotation marks "like this" to indicate direct quotes from your manuscript), or elaborate on this item by providing additional information not in the ms, or briefly explain why the item is not applicable/relevant for your study

"This study investigated a treatment protocol consisting of ICBT modules blended with weekly therapist chat sessions for adolescent depression, including major depressive episode (MDE). We have previously evaluated the treatment in a controlled trial with promising results (d=0.71 against minimal attention control [47]). In line with participant feedback, the protocol was subsequently revised to include longer sessions while the conceptual model of delivery was kept intact. "

"Compared with our previous study [47], the treatment protocol was revised according to participants feedback: sessions were prolonged from 30 to 45 min, texts in some modules were revised to present more clearly (eg, reworked sentences, a more clear description of the rationale for exposure), and information procedures given to participants about weekly themes and goals were standardized."

5-iii) Revisions and updating

Revisions and updating. Clearly mention the date and/or version number of the application/intervention (and comparator, if applicable) evaluated, or describe whether the intervention underwent major changes during the evaluation process, or whether the development and/or content was “frozen” during the trial. Describe dynamic components such as news feeds or changing content which may have an impact on the replicability of the intervention (for unexpected events see item 3b).

	1	2	3	4	5	
subitem not at all important	○	○	○	◉	○	essential

Does your paper address subitem 5-iii?

Copy and paste relevant sections from the manuscript (include quotes in quotation marks "like this" to indicate direct quotes from your manuscript), or elaborate on this item by providing additional information not in the ms, or briefly explain why the item is not applicable/relevant for your study

Your answer

5-iv) Quality assurance methods

Provide information on quality assurance methods to ensure accuracy and quality of information provided [1], if applicable.

	1	2	3	4	5	
subitem not at all important	○	○	○	◉	○	essential

Does your paper address subitem 5-iv?

Copy and paste relevant sections from the manuscript (include quotes in quotation marks "like this" to indicate direct quotes from your manuscript), or elaborate on this item by providing additional information not in the ms, or briefly explain why the item is not applicable/relevant for your study

Your answer

5-v) Ensure replicability by publishing the source code, and/or providing screenshots/screen-capture video, and/or providing flowcharts of the algorithms used

Ensure replicability by publishing the source code, and/or providing screenshots/screen-capture video, and/or providing flowcharts of the algorithms used. Replicability (i.e., other researchers should in principle be able to replicate the study) is a hallmark of scientific reporting.

	1	2	3	4	5	
subitem not at all important	○	○	○	◉	○	essential

Does your paper address subitem 5-v?

Copy and paste relevant sections from the manuscript (include quotes in quotation marks "like this" to indicate direct quotes from your manuscript), or elaborate on this item by providing additional information not in the ms, or briefly explain why the item is not applicable/relevant for your study

Your answer

5-vi) Digital preservation

Digital preservation: Provide the URL of the application, but as the intervention is likely to change or disappear over the course of the years; also make sure the intervention is archived (Internet Archive, webcitation.org, and/or publishing the source code or screenshots/videos alongside the article). As pages behind login screens cannot be archived, consider creating demo pages which are accessible without login.

	1	2	3	4	5	
subitem not at all important	○	○	◉	○	○	essential

Does your paper address subitem 5-vi?

Copy and paste relevant sections from the manuscript (include quotes in quotation marks "like this" to indicate direct quotes from your manuscript), or elaborate on this item by providing additional information not in the ms, or briefly explain why the item is not applicable/relevant for your study

Your answer

5-vii) Access

Access: Describe how participants accessed the application, in what setting/context, if they had to pay (or were paid) or not, whether they had to be a member of specific group. If known, describe how participants obtained “access to the platform and Internet” [1]. To ensure access for editors/reviewers/readers, consider to provide a “backdoor” login account or demo mode for reviewers/readers to explore the application (also important for archiving purposes, see vi).

	1	2	3	4	5	
subitem not at all important	○	○	○	◉	○	essential

Does your paper address subitem 5-vii? *

Copy and paste relevant sections from the manuscript (include quotes in quotation marks "like this" to indicate direct quotes from your manuscript), or elaborate on this item by providing additional information not in the ms, or briefly explain why the item is not applicable/relevant for your study

Access: "Participants were recruited by social media posts from study staff including one guest post in a wide-reaching Instagram account focusing on coping with mental health issues. Posts described the opportunity to receive Web-based psychological treatment within a research study. The treatment included 8 ICBT modules, and 8 individual therapist sessions delivered via chat; the entire intervention lasted 8 weeks. Treatment took place within an encrypted Web-based treatment platform: Iterapi [50]."

Specific group: "Eligible participants were 15 to 19 years, suffered depressive symptoms (≥14 points on the Beck Depression Inventory II; BDI-II [48]), and presented at least 4 symptoms including 1 core symptom, or fulfilled criteria for major depressive episode (MDE) according to The Mini-International Neuropsychiatric Interview (MINI 7.0 [49])."

Compensation: "Participants were not offered financial compensation for treatment or assessment completion at any time."

5-viii) Mode of delivery, features/functionalities/components of the intervention and comparator, and the theoretical framework

Describe mode of delivery, features/functionalities/components of the intervention and comparator, and the theoretical framework [6] used to design them (instructional strategy [1], behaviour change techniques, persuasive features, etc., see e.g., [7, 8] for terminology). This includes an in-depth description of the content (including where it is coming from and who developed it) [1],” whether [and how] it is tailored to individual circumstances and allows users to track their progress and receive feedback” [6]. This also includes a description of communication delivery channels and – if computer-mediated communication is a component – whether communication was synchronous or asynchronous [6]. It also includes information on presentation strategies [1], including page design principles, average amount of text on pages, presence of hyperlinks to other resources, etc. [1].

	1	2	3	4	5	
subitem not at all important	○	○	○	○	◉	essential

Does your paper address subitem 5-viii? *

Copy and paste relevant sections from the manuscript (include quotes in quotation marks "like this" to indicate direct quotes from your manuscript), or elaborate on this item by providing additional information not in the ms, or briefly explain why the item is not applicable/relevant for your study

The treatment included 8 ICBT modules, and 8 individual therapist sessions delivered via chat; the entire intervention lasted 8 weeks. Treatment took place within an encrypted Web-based treatment platform: Iterapi [50]. Modules comprised text material and videos, fictional storylines, reflection tasks and homework assignments, and entailed the behavioral and cognitive approach of CBT. Core techniques included behavioral activation: detecting unhelpful behavior, and reinstating and reinforcing behaviors that increase positive consequences, thus elevating mood [51,52], and cognitive restructuring: correcting maladaptive thinking patterns and inaccurate beliefs (negatively biased views of oneself, of the world in general, and of the future) to reduce depression [53]. Table 1 presents an overview of modules. Therapist chat sessions were coscheduled by participant and therapist each week and were conducted inside the treatment platform. Sessions dealt with the previous and current module and focused on process-related aspects of treatment: identifying problems, examining the patient's cognitions, encouragement, answering questions, and assisting with homework assignments. Participants who agreed were sent reminders before sessions, those who missed a session were offered a new time on the basisof their therapists' remaining availability for the week. In addition to sessions, therapists responded to homework assignments and questions within 24 h on weekdays. The treatment was to be completed on a preset pace of 8 weeks, thereafter participants had access to the program for 4 additional weeks without therapist support.
Compared with our previous study [47], the treatment protocol was revised according to participants feedback: sessions were prolonged from 30 to 45 min, texts in some modules were revised to present more clearly (eg, reworked sentences, a more clear description of the rationale for exposure), and information procedures given to participants about weekly themes and goals were standardized. The overall conceptual model of delivery was kept intact.

Controls were assigned to a therapist and received an introductory personal platform in-mail from their therapist, informing them that there would be weekly assessments and that their assessments were to be viewed by their therapist to monitor their mental health state. They were informed that their therapist might contact them to follow-up on their wellbeing. While being in the control group, participants were allowed to seek regular care, which in Sweden is for free for adolescents.

5-ix) Describe use parameters

Describe use parameters (e.g., intended "doses" and optimal timing for use). Clarify what instructions or recommendations were given to the user, e.g., regarding timing, frequency, heaviness of use, if any, or was the intervention used ad libitum.

	1	2	3	4	5	
subitem not at all important	○	○	○	●	○	essential

Does your paper address subitem 5-ix?

Copy and paste relevant sections from the manuscript (include quotes in quotation marks "like this" to indicate direct quotes from your manuscript), or elaborate on this item by providing additional information not in the ms, or briefly explain why the item is not applicable/relevant for your study

Your answer

5-x) Clarify the level of human involvement

Clarify the level of human involvement (care providers or health professionals, also technical assistance) in the e-intervention or as co-intervention (detail number and expertise of professionals involved, if any, as well as “type of assistance offered, the timing and frequency of the support, how it is initiated, and the medium by which the assistance is delivered”. It may be necessary to distinguish between the level of human involvement required for the trial, and the level of human involvement required for a routine application outside of a RCT setting (discuss under item 21 – generalizability).

	1	2	3	4	5	
subitem not at all important	○	○	○	○	◉	essential

Does your paper address subitem 5-x?

Copy and paste relevant sections from the manuscript (include quotes in quotation marks "like this" to indicate direct quotes from your manuscript), or elaborate on this item by providing additional information not in the ms, or briefly explain why the item is not applicable/relevant for your study

"The treatment included 8 ICBT modules, and 8 individual therapist sessions delivered via chat; the entire intervention lasted 8 weeks."

"Therapist chat sessions were coscheduled by participant and therapist each week and were conducted inside the treatment platform. Compared with our previous study [47], the treatment protocol was revised according to participants feedback: sessions were prolonged from 30 to 45 min. Sessions dealt with the previous and current module and focused on process-related aspects of treatment: identifying problems, examining the patient’s cognitions, encouragement, answering questions, and assisting with homework assignments."

"In addition to sessions, therapists responded to homework assignments and questions within 24 h on weekdays. "

5-xi) Report any prompts/reminders used

Report any prompts/reminders used: Clarify if there were prompts (letters, emails, phone calls, SMS) to use the application, what triggered them, frequency etc. It may be necessary to distinguish between the level of prompts/reminders required for the trial, and the level of prompts/reminders for a routine application outside of a RCT setting (discuss under item 21 – generalizability).

	1	2	3	4	5	
subitem not at all important	○	○	○	○	●	essential

Does your paper address subitem 5-xi? *

Copy and paste relevant sections from the manuscript (include quotes in quotation marks "like this" to indicate direct quotes from your manuscript), or elaborate on this item by providing additional information not in the ms, or briefly explain why the item is not applicable/relevant for your study

"Participants who agreed were sent reminders before sessions."
Additional information: Reminders were sent in the form of text messages

5-xii) Describe any co-interventions (incl. training/support)

Describe any co-interventions (incl. training/support): Clearly state any interventions that are provided in addition to the targeted eHealth intervention, as ehealth intervention may not be designed as stand-alone intervention. This includes training sessions and support [1]. It may be necessary to distinguish between the level of training required for the trial, and the level of training for a routine application outside of a RCT setting (discuss under item 21 – generalizability.

	1	2	3	4	5	
subitem not at all important	○	○	○	●	○	essential

Does your paper address subitem 5-xii? *

Copy and paste relevant sections from the manuscript (include quotes in quotation marks "like this" to indicate direct quotes from your manuscript), or elaborate on this item by providing additional information not in the ms, or briefly explain why the item is not applicable/relevant for your study

Participants received no other than ICBT intervention/control condition.
Therapist received training:
"In total, 6 CBT therapists in training conducted study assessments and treated 5 to 6 ICBT participants and monitored 5 to 6 control participants each. Before assessments, therapists received training in clinician interview (1⁄2 day), ICBT (written material), and how to navigate the treatment platform (1⁄2 day, on demand). Therapists received 60 min of clinical supervision on a weekly basis from clinical psychologists with expertise in adolescent psychopathology and delivery of ICBT."

6a) Completely defined pre-specified primary and secondary outcome measures, including how and when they were assessed

Does your paper address CONSORT subitem 6a? *

Copy and paste relevant sections from the manuscript (include quotes in quotation marks "like this" to indicate direct quotes from your manuscript), or elaborate on this item by providing additional information not in the ms, or briefly explain why the item is not applicable/relevant for your study

"Potential participants were directed to the study website and registered for the study by creating a user account, providing informed consent (checking a box), and completing a Web-based screen (demographics and self-reported outcome measures). An encrypted Web-based treatment platform, Iterapi, was used to collect screening data [50]. "

"The primary outcome was self-reported depression severity at posttreatment, measured by the BDI-II [48]. Secondary outcomes included the MFQ [54]; the beck anxiety inventory (BAI) [55]; the social interaction anxiety scale (SIAS) [56]; the general self-efficacy scale (GSE) [57]; the credibility expectancy questionnaire [58]; the working alliance inventory (WAI-S)[59] and the Brunnsviken Brief Quality of life scale (BBQ) [60]; all self-report scales were completed over Web. The MINI was readministered over phone at posttreatment to assess depression diagnosis, assessors were not blinded to participant allocation at posttreatment."

6a-i) Online questionnaires: describe if they were validated for online use and apply CHERRIES items to describe how the questionnaires were designed/deployed

If outcomes were obtained through online questionnaires, describe if they were validated for online use and apply CHERRIES items to describe how the questionnaires were designed/deployed [9].

	1	2	3	4	5	
subitem not at all important	○	◉	○	○	○	essential

Does your paper address subitem 6a-i?

Copy and paste relevant sections from manuscript text

Outcome measures entail web-based version of established outcomes. However we have not validated outcomes included in this study for online use.

6a-ii) Describe whether and how “use” (including intensity of use/dosage) was defined/measured/monitored

Describe whether and how “use” (including intensity of use/dosage) was defined/measured/monitored (logins, logfile analysis, etc.). Use/adoption metrics are important process outcomes that should be reported in any ehealth trial.

	1	2	3	4	5	
subitem not at all important	○	○	○	◉	○	essential

Does your paper address subitem 6a-ii?

Copy and paste relevant sections from manuscript text

6a-iii) Describe whether, how, and when qualitative feedback from participants was obtained

Describe whether, how, and when qualitative feedback from participants was obtained (e.g., through emails, feedback forms, interviews, focus groups).

	1	2	3	4	5	
subitem not at all important	○	○	○	◉	○	essential

Does your paper address subitem 6a-iii?

Copy and paste relevant sections from manuscript text

Your answer

6b) Any changes to trial outcomes after the trial commenced, with reasons

Does your paper address CONSORT subitem 6b? *

Copy and paste relevant sections from the manuscript (include quotes in quotation marks "like this" to indicate direct quotes from your manuscript), or elaborate on this item by providing additional information not in the ms, or briefly explain why the item is not applicable/relevant for your study

There were no changes to trial outcomes after the trial commenced

7a) How sample size was determined

NPT: When applicable, details of whether and how the clustering by care provides or centers was addressed

7a-i) Describe whether and how expected attrition was taken into account when calculating the sample size

Describe whether and how expected attrition was taken into account when calculating the sample size.

	1	2	3	4	5	
subitem not at all important	○	○	○	◉	○	essential

Does your paper address subitem 7a-i?

Copy and paste relevant sections from manuscript title (include quotes in quotation marks "like this" to indicate direct quotes from your manuscript), or elaborate on this item by providing additional information not in the ms, or briefly explain why the item is not applicable/relevant for your study

Our previous study [47] was used as a reference for power calculations. To detect a similar effect size (Cohen d=0.70) at posttreatment,witha2-tailed5%significancelevelandapower of 80%, a total sample size of 72 was required.

7b) When applicable, explanation of any interim analyses and stopping guidelines

Does your paper address CONSORT subitem 7b? *

Copy and paste relevant sections from the manuscript (include quotes in quotation marks "like this" to indicate direct quotes from your manuscript), or elaborate on this item by providing additional information not in the ms, or briefly explain why the item is not applicable/relevant for your study

We did not perform any interim analyses.

8a) Method used to generate the random allocation sequence

NPT: When applicable, how care providers were allocated to each trial group

Does your paper address CONSORT subitem 8a? *

Copy and paste relevant sections from the manuscript (include quotes in quotation marks "like this" to indicate direct quotes from your manuscript), or elaborate on this item by providing additional information not in the ms, or briefly explain why the item is not applicable/relevant for your study

After consent was agreed and baseline data collected, participants were stratified according to depression severity (fulfilling DSM-5 criteria for MDE or not), and thereafter randomized in a 1:1 ratio. A person not involved in the study executed the randomization procedure using a computer-generated sequence service. Treatment was given open label.

8b) Type of randomisation; details of any restriction (such as blocking and block size)

Does your paper address CONSORT subitem 8b? *

Copy and paste relevant sections from the manuscript (include quotes in quotation marks "like this" to indicate direct quotes from your manuscript), or elaborate on this item by providing additional information not in the ms, or briefly explain why the item is not applicable/relevant for your study

After consent was agreed and baseline data collected, participants were stratified according to depression severity (fulfilling DSM-5 criteria for MDE or not), and thereafter randomized in a 1:1 ratio. A person not involved in the study executed the randomization procedure using a computer-generated sequence service.

9) Mechanism used to implement the random allocation sequence (such as sequentially numbered containers), describing any steps taken to conceal the sequence until interventions were assigned

Does your paper address CONSORT subitem 9? *

Copy and paste relevant sections from the manuscript (include quotes in quotation marks "like this" to indicate direct quotes from your manuscript), or elaborate on this item by providing additional information not in the ms, or briefly explain why the item is not applicable/relevant for your study

After consent was agreed and baseline data collected, participants were stratified according to depression severity (fulfilling DSM-5 criteria for MDE or not), and thereafter randomized in a 1:1 ratio. A person not involved in the study executed the randomization procedure using a computer-generated sequence service. Treatment was given open label.

10) Who generated the random allocation sequence, who enrolled participants, and who assigned participants to interventions

Does your paper address CONSORT subitem 10? *

Copy and paste relevant sections from the manuscript (include quotes in quotation marks "like this" to indicate direct quotes from your manuscript), or elaborate on this item by providing additional information not in the ms, or briefly explain why the item is not applicable/relevant for your study

After consent was agreed and baseline data collected, participants were stratified according to depression severity (fulfilling DSM-5 criteria for MDE or not), and thereafter randomized in a 1:1 ratio. A person not involved in the study executed the randomization procedure using a computer-generated sequence service. Treatment was given open label.

11a) If done, who was blinded after assignment to interventions (for example, participants, care providers, those assessing outcomes) and how

NPT: Whether or not administering co-interventions were blinded to group assignment

11a-i) Specify who was blinded, and who wasn’t

Specify who was blinded, and who wasn’t. Usually, in web-based trials it is not possible to blind the participants [1, 3] (this should be clearly acknowledged), but it may be possible to blind outcome assessors, those doing data analysis or those administering co-interventions (if any).

	1	2	3	4	5	
subitem not at all important	○	○	○	◉	○	essential

Does your paper address subitem 11a-i? *

Copy and paste relevant sections from the manuscript (include quotes in quotation marks "like this" to indicate direct quotes from your manuscript), or elaborate on this item by providing additional information not in the ms, or briefly explain why the item is not applicable/relevant for your study

Treatment was given open label.

11a-ii) Discuss e.g., whether participants knew which intervention was the “intervention of interest” and which one was the “comparator”

Informed consent procedures (4a-ii) can create biases and certain expectations - discuss e.g., whether participants knew which intervention was the “intervention of interest” and which one was the “comparator”.

	1	2	3	4	5	
subitem not at all important	○	○	○	○	◉	essential

Does your paper address subitem 11a-ii?

Copy and paste relevant sections from the manuscript (include quotes in quotation marks "like this" to indicate direct quotes from your manuscript), or elaborate on this item by providing additional information not in the ms, or briefly explain why the item is not applicable/relevant for your study

Your answer

11b) If relevant, description of the similarity of interventions

(this item is usually not relevant for ehealth trials as it refers to similarity of a placebo or sham intervention to a active medication/intervention)

Does your paper address CONSORT subitem 11b? *

Copy and paste relevant sections from the manuscript (include quotes in quotation marks "like this" to indicate direct quotes from your manuscript), or elaborate on this item by providing additional information not in the ms, or briefly explain why the item is not applicable/relevant for your study

There was no similarity between interventions (no sham intervention included in study)

12a) Statistical methods used to compare groups for primary and secondary outcomes

NPT: When applicable, details of whether and how the clustering by care providers or centers was addressed

Does your paper address CONSORT subitem 12a? *

Copy and paste relevant sections from the manuscript (include quotes in quotation marks "like this" to indicate direct quotes from your manuscript), or elaborate on this item by providing additional information not in the ms, or briefly explain why the item is not applicable/relevant for your study

"To detect a similar effect size (Cohen d=0.70) at posttreatment,with a 2-tailed 5% significance level and a power of 80%, a total sample size of 72 was required. A priori: Participants were included in statistical analyses according to the intention-to-treat (ITT) principle. Missing data were handled using multiple imputations. Differences in primary outcomes were evaluated pre- to posttreatment by analysis of covariance (ANCOVA) using baseline values as covariate at the P<.05 level. Effect sizes were calculated based on imputed values and observed standard deviations. Post hoc: Independent t tests and Pearson chi-square tests were used to detect possible baseline differences between groups and percentage decrease in symptoms, respectively. Little missing completely at random test was performed to test the assumption of data missing at random, Levene test was performed to test the assumption of equal variance between groups. Completers were included in complementary statistical analyses for the primary and secondary outcomes. Clinically significant change [61] was determined by investigating the number of participants falling 2 SD below the pretreatment mean for both conditions on the primary outcome, while fulfilling The reliable change index criteria [62], a psychometric criterion used to determine whether an individual change score between baseline and posttreatment assessment is significantly greater than a difference that could have occurred because of random measurement error.

12a-i) Imputation techniques to deal with attrition / missing values

Imputation techniques to deal with attrition / missing values: Not all participants will use the intervention/comparator as intended and attrition is typically high in ehealth trials. Specify how participants who did not use the application or dropped out from the trial were treated in the statistical analysis (a complete case analysis is strongly discouraged, and simple imputation techniques such as LOCF may also be problematic [4]).

	1	2	3	4	5	
subitem not at all important	○	○	○	●	○	essential

Does your paper address subitem 12a-i? *

Copy and paste relevant sections from the manuscript (include quotes in quotation marks "like this" to indicate direct quotes from your manuscript), or elaborate on this item by providing additional information not in the ms, or briefly explain why the item is not applicable/relevant for your study

"Missing data were handled using multiple imputations."

12b) Methods for additional analyses, such as subgroup analyses and adjusted analyses

Does your paper address CONSORT subitem 12b? *

Copy and paste relevant sections from the manuscript (include quotes in quotation marks "like this" to indicate direct quotes from your manuscript), or elaborate on this item by providing additional information not in the ms, or briefly explain why the item is not applicable/relevant for your study

"Post hoc: Independent t tests and Pearson chi-square tests were used to detect possible baseline differences between groups and percentage decrease in symptoms, respectively. Little missing completely at random test was performed to test the assumption of data missing at random, Levene test was performed to test the assumption of equal variance between groups. Completers were included in complementary statistical analyses for the primary and secondary outcomes. Clinically significant change [61] was determined by investigating the number of participants falling 2 SD below the pretreatment mean for both conditions on the primary outcome, while fulfilling The reliable change index criteria [62], a psychometric criterion used to determine whether an individual change score between baseline and posttreatment assessment is significantly greater than a difference that could have occurred because of random measurement error. A criterion of 30% or more increase on the primary outcome from baseline to posttreatment was used to determine significant deterioration."

X26) REB/IRB Approval and Ethical Considerations [recommended as subheading under "Methods"] (not a CONSORT item)

X26-i) Comment on ethics committee approval

	1	2	3	4	5	
subitem not at all important	○	○	○	○	◉	essential

Does your paper address subitem X26-i?

Copy and paste relevant sections from the manuscript (include quotes in quotation marks "like this" to indicate direct quotes from your manuscript), or elaborate on this item by providing additional information not in the ms, or briefly explain why the item is not applicable/relevant for your study

The Research Ethics Board in Linköping, Sweden, gave approval for the study (Reg. no. 2014/427-31). The study was registered at ClinicalTrials.gov (NCT02363205).

x26-ii) Outline informed consent procedures

Outline informed consent procedures e.g., if consent was obtained offline or online (how? Checkbox, etc.?), and what information was provided (see 4a-ii). See [6] for some items to be included in informed consent documents.

	1	2	3	4	5	
subitem not at all important	○	○	○	○	◉	essential

Does your paper address subitem X26-ii?

Copy and paste relevant sections from the manuscript (include quotes in quotation marks "like this" to indicate direct quotes from your manuscript), or elaborate on this item by providing additional information not in the ms, or briefly explain why the item is not applicable/relevant for your study

Potential participants were directed to the study website and registered for the study by creating a user account, providing informed consent (checking a box), and completing a Web-based screen (demographics and self-reported outcome measures). Individuals who showed initial eligibility were invited to a phone interview with study staff to confirm eligibility using the full MINI, to determine matureness to participate, to obtain verbal consent, and to confirm identity (name, address, and personal identity number). Before the randomization, eligible participants were requested to sign a digital consent sheet (full name with digital date stamp) to confirm their willingness to participate in the study.
Study consent were available for the participant and therapist to view at any time inside the web-based platform.

X26-iii) Safety and security procedures

Safety and security procedures, incl. privacy considerations, and any steps taken to reduce the likelihood or detection of harm (e.g., education and training, availability of a hotline)

	1	2	3	4	5	
subitem not at all important	○	○	○	○	○	essential

Does your paper address subitem X26-iii?

Copy and paste relevant sections from the manuscript (include quotes in quotation marks "like this" to indicate direct quotes from your manuscript), or elaborate on this item by providing additional information not in the ms, or briefly explain why the item is not applicable/relevant for your study

A short version of the Mood and Feelings Questionnaire (MFQ-13) and the suicidal ideation item from the Patient Health Questionnaire were used for weekly monitoring of depression. Participants were instructed to immediately contact their therapist in the event of feeling worse and were informed that their therapist might contact them in case of noncompletion. Scores and messages were monitored on a daily basis. In cases of suicidal ideation or significant deterioration, participants were immediately followed up by email and phone. The study collected participants personal identity number and address, and informed participants that in the event of imminent crisis, the study would break confidentiality to pursue appropriate follow-up.

Intervention evaluation: A criterion of 30% or more increase on the primary outcome from baseline to posttreatment was used to determine significant deterioration.

Study reported on negative outcome:
Negative Outcomes
One participant in the ICBT group deteriorated significantly during the course of treatment and was directed to standard care services while being maintained in the study. Post hoc analyses showed that no participant deteriorated significantly following treatment, defined as an increase of 30% or more on the BDI-II from baseline to posttreatment. If those lost to posttreatment assessment were categorized as having deteriorated (n=4), the rate in the ICBT group would be 11%.

RESULTS

13a) For each group, the numbers of participants who were randomly assigned, received intended treatment, and were analysed for the primary outcome

NPT: The number of care providers or centers performing the intervention in each group and the number of patients treated by each care provider in each center

Does your paper address CONSORT subitem 13a? *

Copy and paste relevant sections from the manuscript (include quotes in quotation marks "like this" to indicate direct quotes from your manuscript), or elaborate on this item by providing additional information not in the ms, or briefly explain why the item is not applicable/relevant for your study

Participants were included in statistical analyses according to the intention-to-treat (ITT) principle. Participants randomly assigned, who received intended treatment, and who were analysed for the primary outcome:
ICBT Group: n=35
Control Group: n=35

13b) For each group, losses and exclusions after randomisation, together with reasons

Does your paper address CONSORT subitem 13b? (NOTE: Preferably, this is shown in a CONSORT flow diagram) *

Copy and paste relevant sections from the manuscript (include quotes in quotation marks "like this" to indicate direct quotes from your manuscript), or elaborate on this item by providing additional information not in the ms, or briefly explain why the item is not applicable/relevant for your study

ICBT Group: n=4 lost to post treatment. Missing data were handled using multiple imputations. Study include CONSORT flow diagram (Figure 1).

13b-i) Attrition diagram

Strongly recommended: An attrition diagram (e.g., proportion of participants still logging in or using the intervention/comparator in each group plotted over time, similar to a survival curve) or other figures or tables demonstrating usage/dose/engagement.

	1	2	3	4	5	
subitem not at all important	○	○	○	○	◉	essential

Does your paper address subitem 13b-i?

Copy and paste relevant sections from the manuscript or cite the figure number if applicable (include quotes in quotation marks "like this" to indicate direct quotes from your manuscript), or elaborate on this item by providing additional information not in the ms, or briefly explain why the item is not applicable/relevant for your study

During the period of intervention (8 weeks):
ICBT participants logged into the treatment platform for a mean of 28.4 times (SD 14.6) and completed on average 78% of available modules (mean 6.2 of 8 modules, SD 2.28), and on average 71% of available therapist sessions (mean 5.7 of 8 sessions, SD 2.67). The average total completion was 74% of all 16 available sessions and modules (11.8/16, SD 4.82). In total, 17% participants (6/35) completed less than half of the available treatment modules.

14a) Dates defining the periods of recruitment and follow-up

Does your paper address CONSORT subitem 14a? *

Copy and paste relevant sections from the manuscript (include quotes in quotation marks "like this" to indicate direct quotes from your manuscript), or elaborate on this item by providing additional information not in the ms, or briefly explain why the item is not applicable/relevant for your study

"Enrollment and baseline assessments took place between January 26 and February 10, treatments were conducted between February 13 and April 9, and posttreatment assessments (at 8 weeks) were conducted between April 10 and 16, 2017. Controls were given access to treatment following the posttreatment assessment (8 weeks), and ICBT participants were reassessed 12 months following treatment (April 2018)."

14a-i) Indicate if critical “secular events” fell into the study period

Indicate if critical “secular events” fell into the study period, e.g., significant changes in Internet resources available or “changes in computer hardware or Internet delivery resources”

	1	2	3	4	5	
subitem not at all important	○	○	○	○	○	essential

Does your paper address subitem 14a-i?

Copy and paste relevant sections from the manuscript (include quotes in quotation marks "like this" to indicate direct quotes from your manuscript), or elaborate on this item by providing additional information not in the ms, or briefly explain why the item is not applicable/relevant for your study

Your answer

14b) Why the trial ended or was stopped (early)

Does your paper address CONSORT subitem 14b? *

Copy and paste relevant sections from the manuscript (include quotes in quotation marks "like this" to indicate direct quotes from your manuscript), or elaborate on this item by providing additional information not in the ms, or briefly explain why the item is not applicable/relevant for your study

The trial did not end or was stopped (early).

15) A table showing baseline demographic and clinical characteristics for each group

NPT: When applicable, a description of care providers (case volume, qualification, expertise, etc.) and centers (volume) in each group

Does your paper address CONSORT subitem 15? *

Copy and paste relevant sections from the manuscript (include quotes in quotation marks "like this" to indicate direct quotes from your manuscript), or elaborate on this item by providing additional information not in the ms, or briefly explain why the item is not applicable/relevant for your study

Please see table 2 in manuscript

15-i) Report demographics associated with digital divide issues

In ehealth trials it is particularly important to report demographics associated with digital divide issues, such as age, education, gender, social-economic status, computer/Internet/ehealth literacy of the participants, if known.

	1	2	3	4	5	
subitem not at all important	○	○	○	◉	○	essential

Does your paper address subitem 15-i? *

Copy and paste relevant sections from the manuscript (include quotes in quotation marks "like this" to indicate direct quotes from your manuscript), or elaborate on this item by providing additional information not in the ms, or briefly explain why the item is not applicable/relevant for your study

Manuscript report on participants age, gender, residence (rural, small-town vs city), school study situation (studying, working, drop-out) , family situation (parents, other), parents country of birth. Please se Table 2

16) For each group, number of participants (denominator) included in each analysis and whether the analysis was by original assigned groups

16-i) Report multiple “denominators” and provide definitions

Report multiple “denominators” and provide definitions: Report N’s (and effect sizes) “across a range of study participation [and use] thresholds” [1], e.g., N exposed, N consented, N used more than x times, N used more than y weeks, N participants “used” the intervention/comparator at specific pre-defined time points of interest (in absolute and relative numbers per group). Always clearly define “use” of the intervention.

	1	2	3	4	5	
subitem not at all important	○	○	○	◉	○	essential

Does your paper address subitem 16-i? *

Copy and paste relevant sections from the manuscript (include quotes in quotation marks "like this" to indicate direct quotes from your manuscript), or elaborate on this item by providing additional information not in the ms, or briefly explain why the item is not applicable/relevant for your study

Number consented and included in analyzes described in flowchart (Figure 1).

Effect sizes reported for primary and secondary depression outcome:
"For the primary outcome measure BDI-II, analyses with ANCOVA with baseline scores as covariate revealed a significant effect between groups (F1,67=22.23, P<.001) at posttreatment. The corresponding between-group effect size was d=0.86 (95% CI 0.37-1.35). A post hoc completer analysis (n=66) showed a similar result (d=0.99, 95% CI 0.48-1.51; P<.001). At posttreatment, a significant effect was found for the secondary depression outcome MFQ (F1,67=9.33; P=.003), corresponding to a medium between-group effect of d=0.58 (95% CI 0.10-1.06). A post hoc completer analysis (n=66) showed a similar result (d=0.67; 95% CI 0.18-1.17; P=.002)."

Average use of intervention reported under "program use":
"ICBT participants logged into the treatment platform for a mean of 28.4 times (SD 14.6) and completed on average 78% of available modules (mean 6.2 of 8 modules, SD 2.28), and on average 71% of available therapist sessions (mean 5.7 of 8 sessions, SD 2.67). The average total completion was 74% of all 16 available sessions and modules (11.8/16, SD 4.82). In total, 17% participants (6/35)"

16-ii) Primary analysis should be intent-to-treat

Primary analysis should be intent-to-treat, secondary analyses could include comparing only “users”, with the appropriate caveats that this is no longer a randomized sample (see 18-i).

	1	2	3	4	5	
subitem not at all important	○	○	○	◉	○	essential

Does your paper address subitem 16-ii?

Copy and paste relevant sections from the manuscript (include quotes in quotation marks "like this" to indicate direct quotes from your manuscript), or elaborate on this item by providing additional information not in the ms, or briefly explain why the item is not applicable/relevant for your study

17a) For each primary and secondary outcome, results for each group, and the estimated effect size and its precision (such as 95% confidence interval)

Does your paper address CONSORT subitem 17a? *

Copy and paste relevant sections from the manuscript (include quotes in quotation marks "like this" to indicate direct quotes from your manuscript), or elaborate on this item by providing additional information not in the ms, or briefly explain why the item is not applicable/relevant for your study

Effect sizes reported for primary and secondary depression outcomes:
"For the primary outcome measure BDI-II, analyses with ANCOVA with baseline scores as covariate revealed a significant effect between groups (F1,67=22.23, P<.001) at posttreatment. The corresponding between-group effect size was d=0.86 (95% CI 0.37-1.35). A post hoc completer analysis (n=66) showed a similar result (d=0.99, 95% CI 0.48-1.51; P<.001). At posttreatment, a significant effect was found for the secondary depression outcome MFQ (F1,67=9.33; P=.003), corresponding to a medium between-group effect of d=0.58 (95% CI 0.10-1.06). A post hoc completer analysis (n=66) showed a similar result (d=0.67; 95% CI 0.18-1.17; P=.002). The ANCOVA for posttreatment change in quality of life (BBQ) revealed a significant effect between groups (F1,67=8.73; P=.004). The ANCOVA for pre to posttreatment change in anxiety (BAI) showed no significant effect between groups (F =3.95; P=.051), nor did the ANCOVAs for 1,67 self-efficacy (GSE; P=.81) or social anxiety (SIAS, P=.86)."

Additional information for primary and secondary measures presented in Table 4.

17a-i) Presentation of process outcomes such as metrics of use and intensity of use

In addition to primary/secondary (clinical) outcomes, the presentation of process outcomes such as metrics of use and intensity of use (dose, exposure) and their operational definitions is critical. This does not only refer to metrics of attrition (13-b) (often a binary variable), but also to more continuous exposure metrics such as “average session length”. These must be accompanied by a technical description how a metric like a “session” is defined (e.g., timeout after idle time) [1] (report under item 6a).

	1	2	3	4	5	
subitem not at all important	○	○	○	◉	○	essential

Does your paper address subitem 17a-i?

Copy and paste relevant sections from the manuscript (include quotes in quotation marks "like this" to indicate direct quotes from your manuscript), or elaborate on this item by providing additional information not in the ms, or briefly explain why the item is not applicable/relevant for your study

Your answer

17b) For binary outcomes, presentation of both absolute and relative effect sizes is recommended

Does your paper address CONSORT subitem 17b? *

Copy and paste relevant sections from the manuscript (include quotes in quotation marks "like this" to indicate direct quotes from your manuscript), or elaborate on this item by providing additional information not in the ms, or briefly explain why the item is not applicable/relevant for your study

N/A, effect sizes for binary outcomes not reported.

18) Results of any other analyses performed, including subgroup analyses and adjusted analyses, distinguishing pre-specified from exploratory

Does your paper address CONSORT subitem 18? *

Copy and paste relevant sections from the manuscript (include quotes in quotation marks "like this" to indicate direct quotes from your manuscript), or elaborate on this item by providing additional information not in the ms, or briefly explain why the item is not applicable/relevant for your study

Other pre-specified analyses not performed.

18-i) Subgroup analysis of comparing only users

A subgroup analysis of comparing only users is not uncommon in ehealth trials, but if done, it must be stressed that this is a self-selected sample and no longer an unbiased sample from a randomized trial (see 16-iii).

	1	2	3	4	5	
subitem not at all important	○	○	○	●	○	essential

Does your paper address subitem 18-i?

Copy and paste relevant sections from the manuscript (include quotes in quotation marks "like this" to indicate direct quotes from your manuscript), or elaborate on this item by providing additional information not in the ms, or briefly explain why the item is not applicable/relevant for your study

"A priori: Participants were included in statistical analyses according to the intention-to-treat (ITT) principle. Missing data were handled using multiple imputations." Post hoc: Completers were included in complementary statistical analyses for the primary and secondary outcomes."

19) All important harms or unintended effects in each group

(for specific guidance see CONSORT for harms)

Does your paper address CONSORT subitem 19? *

Copy and paste relevant sections from the manuscript (include quotes in quotation marks "like this" to indicate direct quotes from your manuscript), or elaborate on this item by providing additional information not in the ms, or briefly explain why the item is not applicable/relevant for your study

"Posthoc: A criterion of 30% or more increase on the primary outcome from baseline to posttreatment was used to determine significant deterioration."

"Negative Outcomes
One participant in the ICBT group deteriorated significantly during the course of treatment and was directed to standard care services while being maintained in the study. Post hoc analyses showed that no participant deteriorated significantly following treatment, defined as an increase of 30% or more on the BDI-II from baseline to posttreatment. If those lost to posttreatment assessment were categorized as having deteriorated (n=4), the rate in the ICBT group would be 11%."

19-i) Include privacy breaches, technical problems

Include privacy breaches, technical problems. This does not only include physical "harm" to participants, but also incidents such as perceived or real privacy breaches [1], technical problems, and other unexpected/unintended incidents. "Unintended effects" also includes unintended positive effects [2].

	1	2	3	4	5	
subitem not at all important	○	○	○	○	◉	essential

Does your paper address subitem 19-i?

Copy and paste relevant sections from the manuscript (include quotes in quotation marks "like this" to indicate direct quotes from your manuscript), or elaborate on this item by providing additional information not in the ms, or briefly explain why the item is not applicable/relevant for your study

There were no known privacy breaches or technical problems in this study.

19-ii) Include qualitative feedback from participants or observations from staff/researchers

Include qualitative feedback from participants or observations from staff/researchers, if available, on strengths and shortcomings of the application, especially if they point to unintended/unexpected effects or uses. This includes (if available) reasons for why people did or did not use the application as intended by the developers.

	1	2	3	4	5	
subitem not at all important	○	○	○	◉	○	essential

Does your paper address subitem 19-ii?

Copy and paste relevant sections from the manuscript (include quotes in quotation marks "like this" to indicate direct quotes from your manuscript), or elaborate on this item by providing additional information not in the ms, or briefly explain why the item is not applicable/relevant for your study

Your answer

DISCUSSION

22) Interpretation consistent with results, balancing benefits and harms, and considering other relevant evidence

NPT: In addition, take into account the choice of the comparator, lack of or partial blinding, and unequal expertise of care providers or centers in each group

22-i) Restate study questions and summarize the answers suggested by the data, starting with primary outcomes and process outcomes (use)

Restate study questions and summarize the answers suggested by the data, starting with primary outcomes and process outcomes (use).

	1	2	3	4	5	
subitem not at all important	○	○	○	◉	○	essential

Does your paper address subitem 22-i? *

Copy and paste relevant sections from the manuscript (include quotes in quotation marks "like this" to indicate direct quotes from your manuscript), or elaborate on this item by providing additional information not in the ms, or briefly explain why the item is not applicable/relevant for your study

Objective: "This study investigated a treatment protocol consisting of ICBT modules blended with weekly therapist chat sessions for adolescent depression, including major depressive episode (MDE). We hypothesized that the intervention would outperform the control condition in reducing depression, corresponding to at least moderate between-group effect."

Discussion:
"Principal Findings
We found superiority for the intervention based on the primary outcome measure (BDI-II score at week 8), corresponding to a large between-group effect size (d=0.86). Significant effects on depression symptom level were also observed in the secondary self-reported depression outcome (MFQ, d=0.58), as well as in the number of cases in remission from a major depressive episode (determined using the MINI), further supporting that the intervention was effective. At the 12-month follow-up, depression levels in the treatment group were similar to those observed at posttreatment, follow-up data were not available for the control group. The results for the depression outcomes are in line with, and for the primary outcome surpass, the findings in our previous study that examined the intervention [47]. Although it is important to consider that we made changes to the protocol between studies—foremost, session time was prolonged—the overall conceptual model of delivery was kept intact, and we used comparable design, measures, and procedures across studies. We therefore consider that the findings in this study provide further support for the efficacy of ICBT modules blended with chat sessions to reduce adolescent depression."

Relation to previous research:
"Our protocol included more therapist support than typically found in ICBT. We learned in our previous study that therapist sessions were utilized (completion, mean 78%) but perceived short, and in line with participants' feedback, we prolonged session time in this study. Sessions were completed at a similar degree in this study (mean 71%) with fewer comments on sessions length. Possibly, the extension in time resulted in chat sessions being perceived as sufficient. "
"Although our results are promising and could be associated with the level of therapist support, this was not established by the methods used, and we cannot rule out that other factors have played a role, for example, time to treatment, and initial depression severity. This should be addressed in future studies."
Strengths and limitations:
Strengths of the study include a rigorous design, use of a primary outcome with strong psychometric evidence, and adequate power with regards to detecting between-group differences in the included depression outcomes. ", "A number of limitations should be considered: this study is one of the first on chat-supported ICBT, thus we believed that no evidence-based treatment control group would be appropriate. Nevertheless, it is a limitation that an active control intervention was not included. In line with discussions [21] regarding what is considered an intervention, we moreover labeled our control condition an attention control, as controls were thoroughly assessed and monitored and interacted with. The appropriateness of the label can be discussed. Treatment was open label and participants' awareness of their allocation may have affected self-reported outcomes; similarly, the clinical interviews were not blinded at posttreatment, which calls for caution when interpreting remission rates. ", "Participants in the study were almost entirely female, so results cannot be generalized to males", "Finally, while our positive findings may relate to the novel features of the intervention, the study contributes limited information on how the effects were achieved, for example, on the specific impact of therapist support. Lacking information on what factors influence outcomes in ICBT with youth is a common study limitation [34]."

22-ii) Highlight unanswered new questions, suggest future research

Highlight unanswered new questions, suggest future research.

	1	2	3	4	5	
subitem not at all important	○	○	○	○	○	essential

Does your paper address subitem 22-ii?

Copy and paste relevant sections from the manuscript (include quotes in quotation marks "like this" to indicate direct quotes from your manuscript), or elaborate on this item by providing additional information not in the ms, or briefly explain why the item is not applicable/relevant for your study

Your answer

20) Trial limitations, addressing sources of potential bias, imprecision, and, if relevant, multiplicity of analyses

20-i) Typical limitations in ehealth trials

Typical limitations in ehealth trials: Participants in ehealth trials are rarely blinded. Ehealth trials often look at a multiplicity of outcomes, increasing risk for a Type I error. Discuss biases due to non-use of the intervention/usability issues, biases through informed consent procedures, unexpected events.

	1	2	3	4	5	
subitem not at all important	○	○	○	●	○	essential

Does your paper address subitem 20-i? *

Copy and paste relevant sections from the manuscript (include quotes in quotation marks "like this" to indicate direct quotes from your manuscript), or elaborate on this item by providing additional information not in the ms, or briefly explain why the item is not applicable/relevant for your study

"A number of limitations should be considered"
"Treatment was open label and participants' awareness of their allocation may have affected self-reported outcomes; similarly, the clinical interviews were not blinded at posttreatment, which calls for caution when interpreting remission rates."
"Participants in the study were almost entirely female, so results cannot be generalized to males. Depression is more prevalent among women but gender distributions for Web-based interventions and support can be even more skewed [87,95-96]. There are differences in internet use patterns and preferences between adolescent girls and boys [43], and possibly these differences interact with Web-based interventions as they currently are offered. It has been discussed that young boys are more likely than girls to seek help as a consequence of being influenced by others, and this could explain their relatively low enrollment in Web-based interventions, given that such enrollment is often more dependent on self-motivation and in many cases self-referral [87]. Our study recruited via social media, and moreover via an account focusing on coping with mental health issues; this may have influenced gender uptake as well as attracted particularly motivated participants."
"Finally, while our positive findings may relate to the novel features of the intervention, the study contributes limited information on how the effects were achieved, for example, on the specific impact of therapist support. Lacking information on what factors influence outcomes in ICBT with youth is a common study limitation [34]."

21) Generalisability (external validity, applicability) of the trial findings

NPT: External validity of the trial findings according to the intervention, comparators, patients, and care providers or centers involved in the trial

21-i) Generalizability to other populations

Generalizability to other populations: In particular, discuss generalizability to a general Internet population, outside of a RCT setting, and general patient population, including applicability of the study results for other organizations

	1	2	3	4	5	
subitem not at all important	○	○	○	○	○	essential

Does your paper address subitem 21-i?

Copy and paste relevant sections from the manuscript (include quotes in quotation marks "like this" to indicate direct quotes from your manuscript), or elaborate on this item by providing additional information not in the ms, or briefly explain why the item is not applicable/relevant for your study

Your answer

21-ii) Discuss if there were elements in the RCT that would be different in a routine application setting

Discuss if there were elements in the RCT that would be different in a routine application setting (e.g., prompts/reminders, more human involvement, training sessions or other co-interventions) and what impact the omission of these elements could have on use, adoption, or outcomes if the intervention is applied outside of a RCT setting.

	1	2	3	4	5	
subitem not at all important	○	○	○	○	○	essential

Does your paper address subitem 21-ii?

Copy and paste relevant sections from the manuscript (include quotes in quotation marks "like this" to indicate direct quotes from your manuscript), or elaborate on this item by providing additional information not in the ms, or briefly explain why the item is not applicable/relevant for your study

Your answer

OTHER INFORMATION

23) Registration number and name of trial registry

Does your paper address CONSORT subitem 23? *

Copy and paste relevant sections from the manuscript (include quotes in quotation marks "like this" to indicate direct quotes from your manuscript), or elaborate on this item by providing additional information not in the ms, or briefly explain why the item is not applicable/relevant for your study

"The study was registered at ClinicalTrials.gov (NCT02363205)."

24) Where the full trial protocol can be accessed, if available

Does your paper address CONSORT subitem 24? *

Cite a Multimedia Appendix, other reference, or copy and paste relevant sections from the manuscript (include quotes in quotation marks "like this" to indicate direct quotes from your manuscript), or elaborate on this item by providing additional information not in the ms, or briefly explain why the item is not applicable/relevant for your study

he study was registered at ClinicalTrials.gov (NCT02363205). No study protocol published for this study .

25) Sources of funding and other support (such as supply of drugs), role of funders

Does your paper address CONSORT subitem 25? *

Copy and paste relevant sections from the manuscript (include quotes in quotation marks "like this" to indicate direct quotes from your manuscript), or elaborate on this item by providing additional information not in the ms, or briefly explain why the item is not applicable/relevant for your study

Conflicts of Interest
None declared.

X27) Conflicts of Interest (not a CONSORT item)

X27-i) State the relation of the study team towards the system being evaluated

In addition to the usual declaration of interests (financial or otherwise), also state the relation of the study team towards the system being evaluated, i.e., state if the authors/evaluators are distinct from or identical with the developers/sponsors of the intervention.

	1	2	3	4	5	
subitem not at all important	○	○	○	●	○	essential

Does your paper address subitem X27-i?

Copy and paste relevant sections from the manuscript (include quotes in quotation marks "like this" to indicate direct quotes from your manuscript), or elaborate on this item by providing additional information not in the ms, or briefly explain why the item is not applicable/relevant for your study

Conflicts of Interest
None declared.

About the CONSORT EHEALTH checklist

As a result of using this checklist, did you make changes in your manuscript? *

- ○ yes, major changes
- ○ yes, minor changes
- ○ no

This is a required question

What were the most important changes you made as a result of using this checklist?

Your answer

How much time did you spend on going through the checklist INCLUDING making changes in your manuscript *

3h

As a result of using this checklist, do you think your manuscript has improved? *

- ○ yes
- ○ no
- ◉ Other: already approved

Would you like to become involved in the CONSORT EHEALTH group?

This would involve for example becoming involved in participating in a workshop and writing an "Explanation and Elaboration" document

- yes
- no
- Other:

Any other comments or questions on CONSORT EHEALTH

Your answer

STOP - Save this form as PDF before you click submit

To generate a record that you filled in this form, we recommend to generate a PDF of this page (on a Mac, simply select "print" and then select "print as PDF") before you submit it.

When you submit your (revised) paper to JMIR, please upload the PDF as supplementary file.

Don't worry if some text in the textboxes is cut off, as we still have the complete information in our database. Thank you!

Final step: Click submit !

Click submit so we have your answers in our database!

Submit

Never submit passwords through Google Forms.

This content is neither created nor endorsed by Google. Report Abuse - Terms of Service - Privacy Policy

Google Forms
